# Knowledge, attitudes and practices regarding chemsex prevention among community pharmacy professionals in France: a cross-sectional study

**DOI:** 10.1136/bmjopen-2025-107760

**Published:** 2026-04-17

**Authors:** Matthieu Lebrat, Houssem Eddine Bouasla, Sophie Logerot, Florence Carrouel

**Affiliations:** 1 Hospices Civils de Lyon, Pharmacie et Stérilisation Centrales, F-69000, Lyon, France; 2 Université Lyon 1, P2S, UR 4129, Lyon, France; 3 Grenoble Alpes University, UFR Pharmacie, 38000, Grenoble, France; 4 Grenoble Alpes University, CNRS, UMR 5525, VetAgro Sup, Grenoble INP, TIMC, 38000, Grenoble, France

**Keywords:** General Practice, Surveys and Questionnaires, Sexual and Gender Minorities, Sexually Transmitted Disease, Pharmacists, MENTAL HEALTH

## Abstract

**Abstract:**

**Objectives:**

To describe the Knowledge–Attitude–Practices (KAP) of community pharmacy professionals regarding chemsex prevention in the Auvergne-Rhône-Alpes region of France, and to identify associated determinants.

**Design:**

Regional, cross-sectional, web-based, self-administered KAP survey collecting quantitative and qualitative data, analysed using multivariable linear regression and inductive content analysis.

**Setting:**

Primary care, community pharmacies in the Auvergne-Rhône-Alpes region of France, February–March 2025.

**Participants:**

Of the 276 respondents entering the survey, 261 community pharmacy professionals fully completed it. Inclusion criteria were: (1) age ≥18 years; (2) pharmacists, pharmacy technicians or students in training and (3) employment in a community pharmacy in the Auvergne-Rhône-Alpes region. Exclusion criterion was non-consent.

**Primary and secondary outcome measures:**

KAP domain scores derived from survey items; thematic categories identified through qualitative content analysis of open-ended responses; and factors associated with KAP scores.

**Results:**

Participants were predominantly female (69.6%), pharmacists (64.5%), aged 18–29 (41.7%) and working in urban areas (61.6%). Overall, 67.4% were able to define chemsex. Mean (SD) KAP domain scores were 13.8 (4.4) (range 3–27) for knowledge, 9.6 (2.9) (2–16) for attitudes and 9.6 (2.9) (5–16) for perceived resource adequacy/need. Qualitative analysis identified insufficient knowledge or training (28.7%) and the taboo and intimate nature of the topic (21.1%) as the main barriers to discussing chemsex. The most frequently suggested levers for improving care were professional training (57.1%) and broader prevention efforts (36.4%).

Higher knowledge scores were associated with more positive attitudes (p=0.015), male gender (p=0.004) and prior chemsex-related advice requests (p=0.027), while increasing age was negatively associated with knowledge (p=0.029). Positive attitudes were associated with urban practice settings (p<0.001 for city-centre; p=0.002 for near urban centre) and higher knowledge scores (p<0.001). Perceived resource adequacy/need was lower among participants who had previously provided chemsex-related advice (p=0.047).

**STRENGTHS AND LIMITATIONS OF THIS STUDY**

- Cross-sectional Knowledge–Attitude–Practice (KAP) design generating original baseline data on chemsex prevention in community pharmacies, an underexplored frontline setting.
- Mixed-methods approach combining quantitative analyses with qualitative open-ended responses, providing complementary breadth and contextual insight.
- Use of descriptive statistics and multivariable linear regression to explore associations between KAP dimensions and participant characteristics.
- Non-probability sampling, which may limit representativeness and generalisability.

## Introduction

 Chemsex, a contraction of ‘chemical’ and ‘sex’,^1^ refers to the intentional use of specific psychoactive substances to initiate, intensify or prolong sexual activity, often over extended sessions.^2^ It primarily includes the use of potent psychoactive substances associated with a high risk of dependence, toxicity and sexual health harms, notably methamphetamine, cathinones (mephedrone, 4-methylmethcathinone [4MMC], 3-methylmethcathinone [3MMC]), and gamma-hydroxybutyrate/gamma-butyrolactone (GHB/GBL).

Other substances like alcohol, ketamine, cocaine, poppers and phosphodiesterase type 5 (PDE-5) inhibitors may be used concomitantly in sexualised drug use contexts. However, these substances are generally considered adjunct or co-consumed substances and, when used alone, do not constitute chemsex. Importantly, the mere use of drugs during sex is not sufficient to define chemsex; rather, it is more accurately understood as a distinct behavioural and cultural phenomenon.

According to Stuart D., chemsex is deeply rooted in LGBTQ+ (Lesbian, Gay, Bisexual, Transgender, Queer, and other identities) culture, particularly among men who have sex with men (MSM), and reflects both historical patterns of drug use and evolving sexual practices.^1^ Understanding chemsex therefore requires a sociocultural perspective that considers systemic determinants, such as homophobia, marginalisation, hedonistic subcultures and experiences of interpersonal invalidation.^3^

Sexual health, as defined by the WHO, is a fundamental component of overall health and well-being.^4^ In line with this, France developed a national sexual health strategy (2017–2030) aimed at promoting autonomy, satisfaction and safety in sexual practice, while addressing gender and sexual inequalities that hinder equitable access to sexual health.^5^ This strategy includes chemsex prevention, emphasising the need for primary care professionals to be trained.

While predominantly observed among MSM, chemsex practices have also been reported in other populations.^6^ A 2022 report written for the French Ministry of Health advocates for broadening the definition of chemsex to encompass diverse populations and practices, especially in light of changes following the COVID-19 pandemic.^7^

Estimating the prevalence of chemsex among MSM remains challenging due to heterogenous definitions and study methodologies.^8^ In France, studies conducted between 2015 and 2018 reported prevalence rates ranging from 3% to 21% among MSM.^9 10^ More recent European studies report comparable prevalence estimates, particularly in specific subpopulations. For example, studies conducted in Germany and Sweden reported chemsex prevalences of 14.3% and 18%, respectively, among MSM receiving HIV pre-exposure prophylaxis (PrEP).^11 12^ In addition, a 2025 systematic review and meta-analysis by Georgiadis *et al*, including 238 studies, reported a pooled chemsex prevalence of 22% among MSM, highlighting both the growing body of evidence and the substantial heterogeneity across study contexts.^13^ The increased accessibility of psychoactive substances through geolocated dating applications, such as Grindr, Scruff and Hornet, has likely contributed to the rise in chemsex practices.^14^

Chemsex is associated with several public health challenges, including an increased risk of sexually transmitted infections (STIs) (particularly, HIV),^15^,^18^ serious health complications such as fatal overdoses (with 51 chemsex-related deaths reported in France between 2021 and 2023^2^), as well as significant psychosocial consequences. MSM who engage in chemsex are twice as likely to experience mental health difficulties, including increased symptoms of depression and anxiety.^19 20^ To address these challenges, various community-based and clinical tailored interventions have been developed but rely on strong integrative care with a necessary implication of frontline professionals for screening and first-line treatment.^21^ However, community pharmacies, despite their high accessibility and potential for frontline health promotion, remain largely uninvolved in structured chemsex prevention efforts with no related published data.

Under French law, pharmacists have a legal obligation to contribute to the prevention of drug addiction, as defined in the Public Health Code.^22^ In practice, community pharmacies already serve as a key access point for harm reduction through established interventions such as the dispensation of sterile injection kits (eg, Stéribox) and other public health campaigns (eg, tobacco-free month or Dry January).

Nevertheless, most interactions in pharmacies are still predominantly transactional, centred on product dispensing or brief public health messaging, with limited time and opportunity for in-depth counselling or tailored prevention discussions. Explicitly building on this existing role while moving beyond transactional encounters could strengthen pharmacists’ contribution to chemsex prevention and harm reduction.

Mobilising pharmacists could improve early detection of risky behaviours, facilitate brief interventions and enhance referral pathways within a comprehensive and integrated prevention strategy. In routine pharmacy practice, pharmacists interact with patients primarily through medication dispensing, harm reduction tools and requests for information, rather than through discussions of sexual identity. Their potential role in chemsex prevention therefore lies in responding to substance-related, sexual health-related or medication-related cues (eg, requests for HIV PrEP, postexposure prophylaxis, STI treatment or harm reduction equipment), irrespective of patients’ sexual orientation. This behaviour- and needs-based approach aligns with pharmacists’ professional scope and ethical obligations, while avoiding assumptions about identity.

In order to develop strategies mobilising pharmacists that could combine information provision, harm reduction strategy, effective screening and referral, it is first necessary to generate evidence-based data to inform prevention materials and guidelines. A Knowledge–Attitude–Practice (KAP) study is particularly relevant in this context, as its exploratory approach allows for revealing what a target population knows, believes and how they behave related to a health issue, thereby identifying gaps, misconceptions and informational needs.^23^ With KAP baseline data, prevention tools and policies can be tailored to actual deficits and attitudes with their determinants rather than assumed needs. To date, no chemsex-specific KAP framework has been applied in the pharmacy field, making this exploratory work original.

Therefore, the primary objective of this study was to assess the current level of KAP of community pharmacy health professionals regarding chemsex prevention in the Auvergne-Rhône-Alpes region of France. The secondary objective was to identify determinants associated with these KAP dimensions.

## Materials and methods

### Study design

A cross-sectional KAP survey was conducted to explore community pharmacy professionals’ KAP regarding chemsex prevention. This design was selected because the study objective was exploratory and descriptive, aiming to generate baseline evidence in an area where empirical data are scarce. KAP surveys are commonly used in public health research as a formative approach to identify knowledge gaps, prevailing attitudes and current practices prior to the development of targeted prevention or educational interventions.^23^

Alternative study designs, such as prospective cohort studies or matched control designs, were deemed less appropriate at this stage. Indeed, such designs require predefined exposure–outcome relationships or temporal hypotheses, which were not feasible given the absence of prior data on pharmacists’ involvement in chemsex prevention. Similarly, interventional designs would have been premature without an initial assessment of baseline needs and contextual factors.

To mitigate potential biases inherent to cross-sectional survey designs, several measures were implemented. Participation was voluntary and anonymous to reduce social desirability bias, and the questionnaire employed neutral, non-judgemental wording. Broad dissemination aimed to limit selection bias, and the study adhered to the Checklist for Reporting Of Survey Studies to enhance transparency and methodological rigour^24^ (see online supplemental material S3). While the cross-sectional nature of the study precludes causal inference and temporal assessment, it is well suited to its intended purpose as a preliminary needs assessment informing future intervention design.

This study was conducted between February and March 2025.

### Study population, sampling strategy, recruitment and sample size

This study used a non-probability sampling strategy combining convenience and snowball sampling. The initial sampling frame consisted of community pharmacy training supervisors affiliated with the pharmacy faculties of Grenoble and Lyon, who were contacted via a mailing list. These supervisors were invited to participate and to disseminate the study invitation to colleagues working in community pharmacies. Additional participants were recruited through direct contact and professional networks (word of mouth), further contributing to a convenience-based recruitment approach.

Inclusion criteria were all of the following: (1) adults aged ≥18 years, (2) pharmacists, pharmacy technicians or students in training and (3) currently working in a community pharmacy located in the Auvergne-Rhône-Alpes region of France. Participants meeting all inclusion criteria were eligible for participation. Individuals who did not provide informed consent were excluded.

A minimum sample size of at least 196 participants was targeted to allow estimation of proportions with maximum variance (p=0.50) with a two-sided 95% CI and a margin of error of ±7% (chosen as a pragmatic compromise between acceptable precision and feasibility). The calculation was performed using the pwr package in R (V.4.2.1).

### Questionnaire design and validation

The questionnaire to analyse chemsex prevention KAP was newly developed for this study. As no validated chemsex-specific KAP questionnaire exists to date, general methodological guidelines for KAP survey development and validation were used. It was designed following a literature review conducted by ML and HEB, in accordance with the WHO’s recommendations for KAP survey design^25^ and the methodological framework proposed by Andrade *et al*.^23^ Item generation was informed by the reviewed literature, French public health report on chemsex,^7^ and discussions within the research team. An initial pool of 48 items was generated.

Face and content validity were assessed by an expert committee who independently reviewed items using standardised rating sheets. The expert committee consisted of three pharmacists with experience in community practice and health prevention, and two physicians specialised in addiction medicine and/or mental health, all familiar with chemsex-related issues through clinical or public health practice. Items rated as unsatisfactory were revised or removed, and the final version comprising 31 items was approved by the committee.

As the questionnaire was originally developed in French for a French population, no linguistic translation was required. Cultural relevance and comprehension were addressed through qualitative pretesting.

Cognitive debriefing interviews were conducted with four pharmacists and two final-year pharmacy students to refine clarity, comprehension and relevance of items. During individual interviews, participants were asked to explain their understanding of each item, the meaning attributed to key terms and the rationale for their responses, in line with recommended best practices for questionnaire pretesting.^26^ Feedback from these interviews was used to refine item wording and ensure cultural and contextual appropriateness.

Formal psychometric validation beyond assessment of internal consistency (eg, test–retest reliability or construct validation) was not performed, as the primary objective of this study was exploratory and descriptive.

The final validated questionnaire was hosted on LimeSurvey, accessible online, and required about 5–10 min to complete (online supplemental table S4).

It included 31 questions (25 closed, 6 open-ended), divided into four sections:

- Sociodemographic data (5 questions).
- Chemsex knowledge (14).
- Attitudes toward prevention (6).
- Practices and perceived resource adequacy/needs (6).

### Data collection

Data were collected anonymously via LimeSurvey (dedicated server with 2048-bit SSL data encryption) and exported to R for analysis. No IP addresses or other personally identifiable information were recorded, and age and similar variables were collected in grouped categories to prevent direct or indirect identification. The General Data Protection Regulation (GDPR), the European Union legal framework governing the protection of personal data, was taken into account. As only fully anonymised data were collected, the study complied with GDPR requirements.

### Data analysis

Descriptive statistics were used to summarise participant characteristics and questionnaire responses. Descriptive statistics (percentages) were used for closed-ended items, with means and standard deviations (SD) reported for score variables. All received questionnaires were included in descriptive analysis. For inferential analyses, only complete questionnaires for the relevant variables were included (complete-case analysis). Missing data were not imputed. Missingness primarily resulted from participants discontinuing the questionnaire before completion, as suggested by the pattern of item completion in the received questionnaires, and was therefore assumed to be Missing At Random.

New composite score variables were created:

Knowledge score: Sum of items 6, 7, 9–19 (1 point per correct or valid open-ended response; Likert items scored 0–3). The theoretical score ranged from 0 to 29 points.

Attitude score towards chemsex prevention: Sum of items 21, 23, 26 and 27 (Likert items scored 0–4; specific scoring for item 27 based on agreement with the pharmacists’ role in prevention). The theoretical score ranged from 0 to 13 points.

Perceived resource adequacy/need score (capturing the perceived adequacy of available resources and practical ability to refer and support patients): Sum of items 24, 25, 29 and 30 (Likert items scored 0–4; specific scoring for item 24 based on perceived adequacy of available resources). The theoretical score ranged from 0 to 13 points.

These composite scores were constructed for descriptive and exploratory purposes. Internal consistency was assessed using Cronbach’s alpha; however, the questionnaire was not intended as a fully psychometrically validated scale.

Qualitative free-text responses underwent conventional content analysis.^27^ An inductive approach was applied, allowing categories to emerge directly from the data without a predefined coding framework. Coding was conducted iteratively through familiarisation, open coding and category development, and continued until category saturation was achieved. Meaning units were identified and grouped into categories; multiple categories could be assigned to a single response. Two researchers (ML and HEB) independently coded all responses, with discrepancies resolved through discussion and consensus. Coding decisions were documented to ensure analytic transparency, and representative quotes were selected to illustrate categories. Descriptive frequencies of categories were calculated to identify prominent patterns in the data.

To identify determinants associated with knowledge, attitude, and practice/perceived resource adequacy/need scores, separate multivariable linear regression models were fitted for each outcome. Covariates were selected a priori based on theoretical relevance and previous literature and included sociodemographic characteristics (items 1, 2, 4, 5: profession, pharmacy typology, gender and age), source of awareness about chemsex (item 8), history of chemsex-related advice requests (item 20), and KAP scores.

Years of practice (item 3) were excluded to avoid collinearity with age.

Forward stepwise selection based on the Akaike Information Criterion was then applied as an exploratory variable reduction strategy within this predefined set of covariates. Assumptions of linear regression (linearity, normality of residuals, homoscedasticity and absence of multicollinearity) were assessed using residual plots.

Regression coefficients (β), 95% CIs and p values were reported for all models. A two-sided significance threshold of α=0.05 was used, consistent with conventional practice in exploratory public health research. Given the exploratory nature of the study, the regression analyses were intended to identify potential associations rather than establish causal relationships.

Statistical analyses were performed using R (software V.4.2.1; R Core Team, 2022).

### Patient and public involvement

Patients were not involved in the present study.

## Results

### Sociodemographic data of the community pharmacy health professionals

A total of 276 community pharmacy health professionals responded to the survey from February to March 2025, with 261 fully completed responses (94.6%).

Sociodemographic data are summarised in table 1. Most participants were female (69.6%, n=192/276) pharmacists (64.5%, n=178/276) aged 18 to 29 (41.7%, n=115/276), with less than 10 years of professional experience (53.1%, n=146/275) and the majority reported living in city centres or nearby urban areas (61.6%, n=170/276).

**Table 1:** Sociodemographic characteristics of the community pharmacy professionals

Variable	n	%
Gender		
Male	80	29.0
Female	192	69.6
Unspecified	4	1.4
Profession		
Pharmacist	178	64.5
Pharmacy technician	43	15.6
Student	55	19.9
Age		
18–29	115	41.7
30–44	89	32.2
45–59	50	18.1
60+ years	22	8.0
Years of practice*		
<10 years	146	53.1
10–25 years	75	27.3
>25 years	54	19.6
Pharmacy location		
Rural	106	38.4
City centre	95	34.4
Near an urban centre	75	27.2

n: number of respondents; %: percentage. Total number of respondents: n=276.

* Total number of respondents for this variable was n=275.

Only one missing value was recorded for years of experience and was considered as missing completely at random (MCAR), thus unlikely to introduce bias.

### Knowledge of community pharmacy health professionals about chemsex

The overall knowledge score ranged from 3 to 27, with a mean of 13.8 (SD = 4.4). Cronbach’s alpha indicated acceptable internal consistency for this knowledge score (α=0.77). Most respondents (80.4%, n=222/276) had already heard of chemsex, though only 67.4% (n=186/276) felt able to provide an exact definition. The main source of information was the press or internet (64.1%, n=177/276), with fewer learning through formal education (10.9%, n=30/276) or personal experience (15.2%, n=42/276).

Participants demonstrated mixed understanding of chemsex and its epidemiology. While nearly all recognised the involvement of stimulants such as cocaine, amphetamines and synthetic cathinones (86.2%, n=238/276), fewer correctly identified GHB/GBL and ketamine (60.1%, n=166/276), and many incorrectly included cannabis, LSD or mushrooms (68.1%, n=188/276). Most knew about snorting (89.9%, n=248/276) but only 18.1% (n=50/276) identified ‘slamming’ (intravenous injection).

Knowledge regarding prescription drug use was limited: only 34.4% (n = 95/276) correctly cited examples such as benzodiazepines, pregabalin, amphetamines or PDE-5 inhibitors. Qualitative content analysis showed that opioids were the most frequently cited prescription drugs (21.4%, n=59/276), although they are not typically considered core substances in chemsex.

Regarding drug interactions, 68.8% (n=190/276) felt incapable of managing potential interactions, and only 5.8% (n = 16/276) could name an example. Among the examples provided, the most frequently cited interaction category involved amphetamines or cathinones with selective serotonin reuptake inhibitors (3.3%, n=9/276), which represents a clinically relevant interaction in chemsex contexts. Only four respondents (1.4%) identified a relevant database (Actions Traitements). In contrast, other participants cited regulatory drug information databases (8.3%, n=23/276), which are not specifically designed to address chemsex-related drug–drug interactions.

Regression analysis showed that acquiring knowledge through personal experience was strongly associated with higher knowledge scores in our population (+4.4 points, 95% CI (3.1 to 5.7), p < 0.001). Positive attitudes towards chemsex prevention were also linked to higher knowledge scores (β = 0.2 per attitude point, 95% CI (0.04 to 0.35), p < 0.05). Male gender (+1.5 points, p = 0.004) and previous requests for chemsex-related advice in the pharmacy (+2.5 points, p = 0.027) were additional factors associated with higher knowledge. Conversely, knowledge scores slightly decreased with age (–1.4 points per age group increase, p < 0.05). Online supplemental table S1 summarises these data.

### Attitudes of community pharmacy health professionals towards chemsex prevention

Participants’ attitudes varied widely (mean attitude score = 9.6, SD = 2.9, range: 2–16). Internal consistency of the attitude score was moderate (α=0.61), which is considered acceptable in exploratory KAP research. Nearly half (47.6%, n=124/261) reported moderate or strong apprehension when discussing chemsex at the counter. Qualitative analysis of perceived barriers to discussing chemsex with patients highlighted three main categories: insufficient knowledge or training (28.7%, n=75/261), the taboo and intimate nature of the topic (21.1%, n=55/261) and fear of negative patient reactions (18.8%, n=49/261). Representative quotes illustrating these categories included (translated from French into English by the authors):

«The topic is delicate, and I do not have sufficient knowledge.»

(Student, ID12)

«Fear of making the patient feel uncomfortable*.»*

(Pharmacy technician, ID77)

«Fear of crossing a boundary and facing a negative reaction.»

(Pharmacist, ID173)

About one-quarter (23.7%, n=62/261) agreed with the statement that chemsex users are ‘problematic patients’ reflecting persistent stigma. Nonetheless, 55.6% (n=145/261) believed that pharmacists should play a role in initiating chemsex prevention discussions. Among those who disagreed, the main reasons cited were a perceived misalignment with the pharmacist’s professional role (10.0%, n=26/261) and a lack of appropriate setting and confidentiality (8.4%, n=22/261). Insufficient training or expertise (7.3%, n=19/261) and respect for patient autonomy and private life (6.5%, n=17/261) were also reported.

Representative quotes illustrating these barriers included:

«I think this falls outside the scope of healthcare and our professional competencies.»

(Student, ID5)

«There is very little confidentiality at the counter.»

(Pharmacy technician, ID82)

«Limited training, a need for greater confidentiality than the counter allows, and lack of time.»

(Pharmacist, ID166)

Most participants (75.1%, n=196/261) felt that current prevention approaches in pharmacies inadequately addressed pleasure and social dimensions beyond simple risk information.

Regression analysis showed that working in city centres (+1.5 points, 95% CI (0.71 to 2.3), p < 0.001) or urban peripheries (+1.3 points, 95% CI (0.46 to 2.1), p < 0.05) was associated with higher attitude scores in our population. In addition, higher knowledge scores were positively associated with more favourable attitudes (β = 0.13 per point, p < 0.001). Data are summarised in online supplemental table S2.

### Practices of community pharmacy health professionals regarding chemsex prevention

The perceived resource adequacy/need score had a mean of 9.58 (SD = 2.86, range: 5–16). Cronbach’s alpha indicated good internal consistency for this score (α=0.81). Most participants (86.2%, n = 238/276) had never received a chemsex-related request in their pharmacies. A large majority (83.5%, n=218/261) considered the available information and referral tools for chemsex prevention to be insufficient, and 87.7% rated the overall prevention level for chemsex users as ‘very bad’ or ‘rather bad.’ Despite these gaps, 65.9% felt relatively confident in their ability to refer at-risk individuals to appropriate services. Furthermore, 73.6% indicated they would be willing to support patients with HIV PrEP, vaccination and risk reduction strategies as part of chemsex prevention. Suggestions for improving care were diverse. Following inductive content analysis, several categories emerged. Professional training was the most frequently cited category (57.1%, n=149/261). Other categories included prevention and awareness (broader prevention efforts targeting both the general public and specific populations) (36.4%, n=95/261), information tools and educational materials (34.1%, n=89/261), non-judgemental attitude and destigmatisation (30.7%, n=80/261), orientation and referral to specialised services (26.8%, n=70/261), confidentiality and dedicated consultation spaces (20.3%, n=53/261), screening, identification and early detection (18.4%, n=48/261), multidisciplinary and coordinated care (16.9%, n=44/261), and risk reduction and clinical information (14.9%, n=39/261).

Representative quotes illustrating these levers included:

«More training for pharmacists on chemsex, but also on communication with patients*.* I think there is still a lot to be done, particularly among older generations of pharmacists who, in my view, are not very open to new practices and emerging societal issues such as chemsex or physical and sexual violence.»

(Pharmacist, ID54)

«Community pharmacies should be trained to discuss chemsex in the same way as addictions such as alcohol or tobacco, through prevention campaigns about the associated risks. This would help users feel more legitimate and reassured about turning first to the community pharmacy.»

(Pharmacy technician, ID96)

«Training for healthcare professionals and making the necessary information available to better refer and manage patients (services, consultations, etc.*).»*

(Pharmacist, ID132)

«Offering more regular training to healthcare professionals, including pharmacy technicians, to help them identify at-risk patients and address the topic with them. Talking about chemsex so that it is no longer taboo among healthcare professionals in general. Establishing a set of criteria and questions to screen at-risk patients, along with a list of specialized services that can support them if problems arise from this practice.»

(Student, ID187)

«TRAIN HEALTHCARE PROFESSIONALS. Public prevention campaigns. Systematic information provided by healthcare professionals during sexually transmitted infections screening (physicians, laboratories, testing centers). Providing information when dispensing antibiotics for sexually transmitted infections, PrEP, or prescriptions related to sexual health (particularly for MSM).»

(Pharmacist, ID245)

Interestingly, having previously received a chemsex-related request was associated with a lower perceived resource adequacy/need in our population (–1.2 points, 95% CI (–2.4 to –0.01), p = 0.047), suggesting that direct exposure may improve perceived preparedness. Data are summarised in table 2.

**Table 2:** Practices and perceived resource adequacy/needs of community pharmacy health professionals about chemsex prevention and its determinants

Variable		n	%
Received chemsex-related advice requests*	Yes	12	4.4
	No	238	86.2
	Do not know	26	9.4
Perceived adequacy of chemsex-related informational and referral tools for pharmacy staff	Yes	6	2.3
	No	218	83.5
	Do not know	37	14.2
Perceived adequacy of prevention information available to chemsex users	Very good	1	0.4
	Rather good	2	0.8
	Neutral	29	11.1
	Rather bad	124	47.5
	Very bad	105	40.2
Self-perceived ability to refer patients at risk of addiction or vulnerability to appropriate services	Very unlikely	16	6.1
	Rather unlikely	48	18.4
	Don’t know	25	9.6
	Rather likely	135	51.7
	Very likely	37	14.2
Self-perceived ability to support chemsex users with PrEP, vaccination and infection risk reduction	Very unlikely	5	1.9
	Rather unlikely	24	9.2
	Don’t know	40	15.3
	Rather likely	144	55.2
	Very likely	48	18.4
Participants’ suggestions for improving care of patients engaging in chemsex	(Free text) Categories that emerged after inductive content analysis ‡:		
	Professional training	149	57.1
	Prevention and awareness	95	36.4
	Information materials	89	34.1
	Non-judgement and destigmatisation	80	30.7
	Referral to specialised care	70	26.8
	Confidential consultations	53	20.3
	Screening and identification	48	18.4
	Multidisciplinary care	44	16.9
	Risk reduction	39	14.9
Perceived resource adequacy/need score*			
**Mean**	**SD**	**Min**	**Max**
9.58	2.86	5	16
**Linear multivariable regression assessing the determinants of the perceived adequacy of resources and needs for chemsex prevention among professionals**			
**Variable** †	**Beta (β**)	**95%** **CI**	**P value**
Age	0.18	−0.46 to 0.81	0.6
Received chemsex-related advice requests			
No	–	–	Reference
Yes	−1.2	−2.4 to −0.01	0.047*
Do not know	0.48	−1.7 to 0.74	0.4

n: number of respondents, %: percentage. SD: Standard deviation. CI: Confidence Interval. The total number of respondents was 261.

Beta (β) is the regression coefficient and can be interpreted as a change in outcome variable per unit change in predictor.

For p-values, a single asterisk (*) indicates statistical significance at p < 0.05, and a double asterisk (**) indicates statistical significance at p < 0.001.

* Total number of respondents for this variable was n=276.

† Variables after this table line were included in the final mdel. Multiple R-squared: 0.08. Age was modelled as an ordinal variable (four categories: 18–29, 30–44, 45–59, 60+ years), and the linear contrast was used to capture a trend across increasing age groups.

‡ Categories are not mutually exclusive because responses could address multiple themes

PrEP, pre-exposure prophylaxis.

## Discussion

Chemsex is still disseminating across vulnerable populations in many countries, including France, and remains a public health challenge. Community pharmacies ensure easy access to care through their territorial network. They contribute to prevention, making interventions regarding addiction particularly valuable in care pathways. This study described the KAP of community pharmacy health professionals in Auvergne-Rhône-Alpes and identified associated determinants to inform the development of training for these professionals and strengthen their potential contributions to chemsex prevention.

Overall, our findings highlight heterogeneous levels of knowledge and significant gaps among participants. While many professionals recognised the substances typically involved in chemsex, few reported specific knowledge related to chemsex such as feeling capable of managing drug interactions or identifying patient vulnerability. Furthermore, the substantial proportion of participants who incorrectly endorsed cannabis or hallucinogens as chemsex-related substances suggests a broader interpretation of chemsex as ‘drug use during sex’, rather than the more specific set of substances commonly emphasised in chemsex-related harm profiles. This misclassification underscores the need for clearer definitional and educational training. These limited knowledge levels align with previous studies conducted in Spain and elsewhere, which have also reported low chemsex awareness among primary care professionals.^28^ Only 10.9% of surveyed participants reported having learnt about chemsex through initial training or continuing education, highlighting a substantial educational gap and supporting the need to strengthen chemsex-related content in pharmacy curricula and continuing professional development programmes. Our results from our studied population further suggest that higher knowledge scores were significantly associated with positive attitudes (p=0.015), male gender (p=0.004) and prior requests for chemsex-related advice (p=0.027), while increasing age was negatively associated (p=0.029).

The association between male gender and higher knowledge scores should be interpreted cautiously and does not imply inherent gender-based differences. Several alternative mechanisms may account for this finding, including differential exposure to information on chemsex through professional encounters, continuing education or informal networks; greater likelihood of prior contact with chemsex-related situations; or differences in self-perceived confidence when responding to knowledge items. Workplace role distribution and task allocation within pharmacies may also influence exposure to sexual health or harm reduction topics. Given the cross-sectional and self-reported nature of the data, residual confounding and reporting bias cannot be excluded. Additionally, professionals who had previously provided chemsex-related guidance may have developed greater awareness through direct experience, although the direction of this relationship cannot be determined with this study. The negative association with age may reflect generational differences in training exposure on this relatively recent public health issue, although alternative explanations cannot be excluded. Similar patterns have been observed in other KAP studies, such as those focusing on HPV vaccination, where knowledge declined with professional experience.^29^

Regarding knowledge gaps with direct implications for harm-reduction counselling, the low recognition of injection (‘slam’) as part of chemsex (18.1%) is particularly concerning. This limited awareness may restrict pharmacists’ ability to deliver appropriate harm-reduction messages when dispensing sterile injection equipment, despite the elevated risks of acute toxicity and bloodborne infections associated with injecting practices. Given that community pharmacies already serve as a key access point for sterile equipment, insufficient recognition of slam may result in missed opportunities for targeted counselling and risk reduction.

In addition, knowledge gaps regarding drug interactions remain concerning. Many chemsex substances are metabolised by the CYP450 system, and interactions with antiretroviral or psychoactive medications can carry serious risks, underscoring the need for better training and reference tools. Moreover, much of the current evidence relies on case reports.^30^ Studies on these specific interactions should be undertaken.

At the policy level, these findings suggest that integrating basic chemsex-related content into pharmacy undergraduate curricula and continuing professional development, as well as developing practical reference tools for community pharmacies, may represent feasible and proportionate first steps to strengthen prevention, particularly given the low baseline exposure observed in this study.

Regarding attitudes, just over half of the professionals (55.6%) felt that pharmacists should engage in chemsex prevention. However, nearly half reported apprehension discussing this topic, citing insufficient knowledge or training (28.7%), taboo and intimate nature of the topic (21.1%) or fear of negative patient reactions (18.8%) as barriers.

Notably, 17.2% (n=45/261) of respondents believed it was not their responsibility, despite legal and ethical obligations for pharmacists to contribute to addiction prevention.^22 31^

Positive attitudes towards chemsex prevention were associated in our population with working in urban areas (p<0.001 in city centres, p=0.002 in urban peripheries) and higher knowledge scores (p<0.001). This may be explained by a greater prevalence of chemsex in urban areas,^32^ potentially increasing exposure and fostering more supportive attitudes, but causal inferences cannot be drawn. Chemsex also occurs in rural settings, suggesting that professionals everywhere should be trained.^33^

In practice, very few professionals reported having direct experience providing chemsex-related advice (4.4%). At the same time, most participants perceived prevention efforts and available tools as insufficient, highlighting a lack of structured resources and guidance. Despite this, many reported feeling able to refer at-risk patients and expressed willingness to support related services such as HIV PrEP, vaccination and STI risk reduction, indicating a foundation on which to build.

Although this contrast may appear contradictory, it likely reflects the distinction between general referral capacity and chemsex-specific preparedness. Referral to sexual health or addiction services often relies on existing, well-established pathways with which pharmacy professionals are already familiar, whereas chemsex-specific prevention requires tailored knowledge, tools and training that are currently lacking. The discrepancy between low demonstrated knowledge of drug–drug interactions or reference resources and high self-reported referral capacity further suggests that professionals may conceptualise chemsex primarily as a psychosocial issue requiring referral, potentially overlooking medication-safety responsibilities at the point of dispensing. Training initiatives could therefore benefit from integrating both referral pathways and practical medication-safety decision support. In addition, social desirability bias may have contributed to over-reporting perceived referral capacity. Interestingly, having previously received chemsex-related questions was negatively associated with the perceived adequacy of resources and needs score (p=0.047), which may reflect differences in perceived preparedness among professionals with prior exposure; however, reverse causality and unmeasured confounding are possible. Overall, these findings highlight the importance of targeted training and practical tools to bridge the gap between willingness to act and effective chemsex-specific prevention practices.

Our findings emphasise the necessity of enhancing chemsex-related education among community pharmacy staff. Given the positive link between knowledge and attitudes observed in our study, strengthening educational resources may encourage greater engagement in prevention activities. Information has indeed been identified as a key component of support and prevention programmes for chemsex users; therefore, improving professionals’ awareness is essential to further develop patient education.^8^ However, any educational approach should consider professionals’ fears and patients’ barriers in disclosing sexual health concerns, as observed in contexts like PrEP discussions.^34^ Fear of judgement and shame has been identified in multiple studies among chemsex users and may discourage them from seeking care.^835^,^37^ In our study, qualitative findings also highlighted professionals’ apprehension in discussing chemsex, largely related to lack of knowledge, taboo, stigma and concerns about patient reactions. On the other hand, surveyed professionals identified a non-judgemental and destigmatisation approach as a potential lever for improving care of patients engaging in chemsex. Together, these findings support the development of non-judgemental and destigmatising prevention strategies. A patient-centred approach, developed in partnership with community organisations and local services, is essential to ensure culturally sensitive and effective interventions.

### Strengths and limitations

The study has notable strengths. To our knowledge, this is the first investigation of KAP regarding chemsex prevention among community pharmacy health professionals, not only in France but internationally. With 261 fully completed responses, the study provides robust baseline data. The use of a structured KAP framework, combined with both quantitative analyses and qualitative content analysis of open-ended responses, offers a comprehensive and nuanced understanding of current gaps, perceptions and needs relevant to chemsex prevention in community pharmacy settings. In addition, internal consistency of the composite scores was acceptable to good (Cronbach’s alpha: knowledge=0.77; attitude=0.61; perceived resource adequacy/need=0.81), supporting the coherence of the constructs measured for exploratory purposes.

Nevertheless, this study has several limitations. The cross-sectional design prevents causal conclusions. Potential selection bias exists with the use of convenience and snowball sampling. Participants with a particular interest in sexual health or harm reduction may have been more likely to respond, as well as those more engaged with continuing education or university networks, since the initial sampling frame was derived from pharmacy training supervisors affiliated with the faculties of Grenoble and Lyon, which may have led to an overrepresentation of professionals connected to these academic networks. Consequently, observed knowledge gaps may represent a conservative estimate relative to the broader community pharmacy workforce, with findings that may not be fully generalisable to all community pharmacy professionals in the Auvergne-Rhône-Alpes region or elsewhere in France. However, cautious interpretation is warranted, as the sample shares several characteristics with the national pharmacy workforce (including a predominance of female pharmacists^38^) and includes professionals from diverse professional backgrounds and age groups.

Additional sources of bias and confounding should also be considered. Variables such as prior training in sexual health or addiction, workload, pharmacy size, socioeconomic characteristics of the catchment area, and frequency of exposure to key populations (eg, MSM or PrEP users) were not measured and may have influenced both knowledge and attitudes toward chemsex prevention. Consequently, residual confounding cannot be excluded. Information bias may have occurred due to self-reported data, including recall bias regarding prior chemsex-related requests and social desirability bias, particularly for attitudes and perceived practices related to stigmatised behaviours. Participants may have over-reported favourable attitudes toward prevention or perceived preparedness. To mitigate this risk, the questionnaire was anonymous, self-administered and completed online, with no identifying information collected. Despite face and content validation and cognitive debriefing during questionnaire development, some measurement error related to item framing or response scales cannot be entirely excluded.

The multivariable regression models explained a moderate variance for knowledge (35.8%) but less for attitudes (11%) and perceived resource adequacy/need (8%). This suggests that while individual characteristics and prior exposure to chemsex-related situations partly explain knowledge levels, attitudes and perceived practices are likely shaped by additional unmeasured factors. These may include personal beliefs, perceived professional role boundaries, prior trainings, moral or cultural representations of sexuality and substance use, prior informal experiences with chemsex-related situations, organisational context and perceived institutional support. Moreover, the use of forward stepwise regression may have introduced model instability or overfitting, and the identified associations should therefore be interpreted as exploratory and hypothesis-generating rather than confirmatory.

Finally, although internal consistency of the composite scores was acceptable, the questionnaire was newly developed and not intended as a fully psychometrically validated instrument. The Attitude score showed moderate reliability (Cronbach’s α=0.61), which is acceptable for exploratory research but suggests that further refinement and validation are warranted. Further studies should aim to confirm these findings and to conduct comprehensive psychometric validation of chemsex-specific KAP tools for pharmacy professionals.

## Conclusions

These findings highlight the need for training community pharmacy staff in chemsex prevention to strengthen their primary care role. Participants expressed interest in resources to improve knowledge and support users through a positive, non-stigmatising, patient-centred approach. Training should involve experts and community organisations to integrate prevention effectively. Our findings could inform chemsex prevention policies and strategies in the community pharmacy setting.

Future research should explore patient needs and rigorously evaluate the design of prevention interventions to guide wider implementation in healthcare systems.

## Supplementary material

10.1136/bmjopen-2025-107760 online supplemental file 1

10.1136/bmjopen-2025-107760 online supplemental file 2

10.1136/bmjopen-2025-107760 online supplemental file 3

10.1136/bmjopen-2025-107760 online supplemental file 4

## Data Availability

Data are available on reasonable request.
