# Supplementary material for: Knowledge, attitudes and practices regarding chemsex prevention among community pharmacy professionals in France: a cross-sectional study
Source: BMJ Open. 2026 Apr 17;16(4):e107760. doi: 10.1136/bmjopen-2025-107760 (PMC13110612; doi:10.1136/bmjopen-2025-107760)
Supplement: online supplemental file 1 [file bmjopen-16-4-s001.docx]

**S1 Table. Knowledge of community pharmacy health professionals about chemsex and its determinants**

| **Variable** | | **n** | **%** |
| --- | --- | --- | --- |
| *Chemsex awareness prior to this study* | Yes | 222 | 80.4 |
| *Self-declared ability to define chemsex* | Yes | 186 | 67.4 |
| *Means of knowledge about chemsex* | initial training or continuing education | 30 | 10.9 |
|  | through the press, literature, or the internet | 177 | 64.1 |
|  | personally | 42 | 15.2 |
|  | Other means [Free text]  Categories that emerged after inductive content analysis:   - Self-initiated information seeking due to the survey - Media coverage of high-profile case - From pharmacy staff discussions - Formal education training in addictology - From patients - From dating apps | 28  11  6  6  2  2  1 | 10.1  4.0  2.2  2.2  0.7  0.7  0.4 |
| *Epidemiology* | Between 2021 and 2023, more than 500 cases of chemsex-related complications  were reported to the Centres d'Evaluation et d'Information sur la Pharmacodépendance-Addictovigilance (CEIP-A) in France | 191 | 69.2 |
|  | We are witnessing a decline in infectious complications among chemsex users | 270 | 97.8 |
|  | In France, the number of people potentially affected by chemsex is estimated at between 100,000 and 200,000 | 165 | 59.8 |
|  | The Décès en Relation avec l'Abus de Médicaments Et de Substances (DRAMES) survey conducted by the CEIP-A in France lists more than 50 chemsex-related deaths between 2021 and 2022 | 175 | 63.4 |
|  | 90% of deaths are directly linked to intoxication by synthetic cathinones (3-MMC, 4-MMC...etc) | 103 | 37.3 |
|  | Chemsex affects the general population regardless of gender or sexual orientation | 193 | 69.9 |
|  | Chemsex is more prevalent among men who have sex with men (MSM) | 186 | 67.4 |
|  | Chemsex may be more prevalent among PrEP users (Pre-Exposure Prophylaxis for HIV, Emtricitabine / Tenofovir) | 114 | 41.3 |
|  | Chemsex may also affect non-MSM people | 189 | 68.5 |
| *Drugs involved in Chemsex* | Cannabis/LSD/Hallucinogenic mushrooms | 188 | 68.1 |
|  | GHB/GBL/Ketamine | 166 | 60.1 |
|  | Cocaine, amphetamines and synthetic cathinones: 3 MMC, 4 MMC | 238 | 86.2 |
| *Drug consumption methods* | Snorting | 248 | 89.9 |
|  | Swallow | 132 | 47.8 |
|  | Slam | 50 | 18.1 |
|  | Plug or booty-bumping | 95 | 34.4 |
| *Prescription molecules used in Chemsex* | At least one correct answer cited [Free text]  Categories that emerged after inductive content analysis: (true or false responses) (2):   - Opioids - Benzodiazepines - Phosphodiesterase type 5 (PDE-5) inhibitors - Pregabalin - Tramadol - Sympathomimetic agents - Ketamine | 95  59  49  28  18  16  15  3 | 34.4  21.4  17.8  10.1  6.5  5.8  5.4  1.1 |
| *Self-declared ability to manage interactions between chemsex drugs and medicines* | Very capable | 0 | 0 |
|  | Somewhat capable | 2 | 0.7 |
|  | Don't know | 21 | 7.6 |
|  | Somewhat incapable | 63 | 22.8 |
|  | Incapable | 190 | 68.8 |
| *Ability to cite an interaction between drugs and medicines* | At least one correct answer cited [Free text]  Categories that emerged after inductive content analysis: (true or false responses) (2):   - Amphetamine and Cathinones – SSRIs - Poppers – PDE-5 inhibitors - HIV-drugs related interactions - Tramadol - SSRIs - Benzodiazepines – GHB/GBL - Opioids - Beta_2_-adrenergic agonists - Benzodiazepines – Cathinones - GHB/GBL – PDE-5 inhibitors | 16  9  3  3  3  1  1  1  1 | 5.8  3.3  1.1  1.1  1.1  0.4  0.4  0.4  0.4 |
|  | None | 260 | 94.2 |
| Self-declared ability to cite a drug interaction database relevant to chemsex | Yes | 26 | 9.4 |
| *Ability to cite a database for chemsex drug-related interactions* | At least one correct answer cited  Categories that emerged after inductive content analysis: (true or false responses):   - Regulatory drug information databases - Specific chemsex database (“Actions Traitements”) | 4  23  4 | 1.4  8.3  1.4 |
|  | None | 272 | 98.6 |
| *Therapeutic adherence* | Chemsex can have an impact on some patients' adherence to their treatments | 195 | 70.7 |
| *Self-declared ability to identify signs of vulnerability or impacts of chemsex* | Unable or don’t know | 134 | 48.6 |
|  | Somewhat unable | 65 | 23.6 |
|  | Somewhat able | 74 | 26.8 |
|  | Able | 3 | 1.1 |
| **Knowledge score*** | | | |
| **mean** | **standard deviation** | **min** | **max** |
| 13.81 | 4.42 | 3 | 27 |
| **Linear multivariable regression performed to assess the determinants of knowledge regarding chemsex and its prevention** | | | |
| **Variable (1)** | **Beta (β)** | **95% CI** | **p-value** |
| **Means of knowledge about chemsex** |  |  |  |
| initial training or continuing education | 2.8 | 1.3, 4.2 | <0.001** |
| through the press, literature, or the internet | 3.2 | 2.2, 4.2 | <0.001** |
| personally | 4.4 | 3.1, 5.7 | <0.001** |
| **Attitude score** | 0.2 | 0.04, 0.35 | 0.015* |
| **Gender** |  |  |  |
| woman | - | - | Reference |
| man | 1.5 | 0.49, 2.5 | 0.004* |
| undisclosed or other | 1.9 | -2.3, 6.2 | 0.4 |
| **Received chemsex-related advice requests** |  |  |  |
| no | - | - | Reference |
| yes | 2.5 | 0.29, 4.7 | 0.027* |
| do not know | 1.4 | -0.82, 3.7 | 0.2 |
| **Age** | -1.4 | -2.6, -0.14 | 0.029* |
| Note: The results are expressed as n (%). n: number of respondents, %: percentage. Total number of respondents was n= 276. * For the created variable “knowledge score”, only complete questionnaires were considered (n= 261). (1) Variables after this table line were included in the final model. Multiple R-squared: 0.36. Age was modeled as an ordinal variable (four categories: 18–29, 30–44, 45–59, 60+ years), and the linear contrast was used to capture a trend across increasing age groups. CI: Confidence Interval. Beta (β) is the regression coefficient and can be interpreted as a change in outcome variable per unit change in predictor. For p-values, a single asterisk (*) indicates statistical significance at p < 0.05, and a double asterisk (**) indicates statistical significance at p < 0.001.  (2): Categories are not mutually exclusive because responses could address multiple themes | | | |
