# Supplementary material for: Knowledge, attitudes and practices regarding chemsex prevention among community pharmacy professionals in France: a cross-sectional study
Source: BMJ Open. 2026 Apr 17;16(4):e107760. doi: 10.1136/bmjopen-2025-107760 (PMC13110612; doi:10.1136/bmjopen-2025-107760)
Supplement: online supplemental file 2 [file bmjopen-16-4-s002.docx]

**S2 Table. Attitude of community pharmacy health professionals about chemsex prevention and its determinants**

| **Variable** | | | **n** | **%** |
| --- | --- | --- | --- | --- |
| *Comfort discussing chemsex with at-risk patients* | No apprehension | | 41 | 15.7 |
|  | Rather no apprehension | | 55 | 21.1 |
|  | Neutral | | 41 | 15.7 |
|  | Moderate apprehension | | 74 | 28.4 |
|  | Strong apprehension | | 50 | 19.2 |
| *Barriers to discussing chemsex with patients* | [Free text]  Categories that emerged after inductive content analysis (2):   - Insufficient knowledge/training - Taboo and intimacy of the topic - Fear of negative patient reactions - Lack of confidentiality - Fear of stigmatization or judgment - Difficulty raising the topic - Patient/contextual factors - Legal concerns | 75  55  49  30  28  23  18  10 | | 28.7  21.1  18.8  11.5  10.7  8.8  6.9  3.8 |
| *Perception of chemsex patients as problematic* | Strongly agree | | 9 | 3.4 |
|  | Somewhat agree | | 53 | 20.3 |
|  | Neither agree nor disagree | | 102 | 39.1 |
|  | Somewhat disagree | | 54 | 20.7 |
|  | Strongly disagree | | 43 | 16.5 |
| *Approach to chemsex prevention: addressing pleasure and social aspects*  *[Participants had to tell if they agree this approach is sufficiently addressed]* | Strongly agree | | 4 | 1.5 |
|  | Somewhat agree | | 14 | 5.4 |
|  | Neither disagree nor agree | | 47 | 18.0 |
|  | Somewhat disagree | | 122 | 46.7 |
|  | Strongly disagree | | 74 | 28.4 |
| *Pharmacist's role in initiating chemsex discussions for prevention* | Yes | | 145 | 55.6 |
|  | No  Reason for this answer: [Free text]  Categories that emerged after inductive content analysis (2):   - Perceived misalignment with the pharmacist’s professional role - Lack of appropriate setting and confidentiality - Insufficient training or expertise - Respect for patient autonomy and private life - Time constraints and lack of recognition | | 45  26  22  19  17  11 | 17.2  10.0  8.4  7.3  6.5  4.2 |
|  | Do not know | | 71 | 27.2 |
| **Attitude score** | | | | |
| **mean** | **standard deviation** | | **min** | **max** |
| 9.57 | 2.86 | | 2 | 16 |
| **Linear multivariable regression performed to assess the determinants of the attitude score towards chemsex prevention** | | | | |
| **Variable (1)** | **Beta (β)** | | **95% CI** | **p-value** |
| **Pharmacy location** |  | |  |  |
| rural | - | | - | Reference |
| city center | 1.5 | | 0.71, 2.3 | <0.001** |
| near an urban center | 1.3 | | 0.46, 2.1 | 0.002* |
| **Knowledge score** | 0.13 | | 0.05, 0.21 | <0.001** |
| **Means of knowledge about chemsex** |  | |  |  |
| through the press, literature, or the internet | -0.54 | | -1.2, 0.18 | 0.14 |
| Note: The results are expressed as n (%). n: number of respondents, %: percentage. The total number of respondents was 261. (1) Variables after this table line were included in the final model. Multiple R-squared: 0.11. CI: Confidence Interval. Beta (β) is the regression coefficient and can be interpreted as a change in outcome variable per unit change in predictor. For p-values, a single asterisk (*) indicates statistical significance at p < 0.05, and a double asterisk (**) indicates statistical significance at p < 0.001.  (2): Categories are not mutually exclusive because responses could address multiple themes | | | | |
