# Supplementary material for: Knowledge, attitudes and practices regarding chemsex prevention among community pharmacy professionals in France: a cross-sectional study
Source: BMJ Open. 2026 Apr 17;16(4):e107760. doi: 10.1136/bmjopen-2025-107760 (PMC13110612; doi:10.1136/bmjopen-2025-107760)
Supplement: online supplemental file 4 [file bmjopen-16-4-s004.docx]

**S4 Table. Questionnaire.** Survey questionnaire (Original French version)

| **N° de question** | **Question** | **Réponse** |
| --- | --- | --- |
| 1 | Quel est votre métier ? | - Pharmacien (Titulaire, Adjoint, Salarié)  - Préparateur  - Étudiant en Pharmacie ou Étudiant préparateur |
| 2 | Dans quel type de pharmacie travaillez-vous actuellement ? | - Rural ou semi-rural  - Centre-ville  - En périphérie d’une grande ville ou d’un centre urbain |
| 3 | Depuis combien de temps pratiquez-vous l’exercice pharmaceutique ? (Après obtention du diplôme) | - Depuis moins de 10 ans  - Depuis 10 ans à 25 ans  - Depuis plus de 25 ans |
| 4 | Quel est votre sexe ? | - Femme  - Homme  - Ne souhaite pas préciser / Autre |
| 5 | Quel est votre âge ? | - Entre 18 et 29 ans  - Entre 30 et 44 ans  - Entre 45 et 59 ans  - 60 ans et plus |
| 6 | Avez-vous déjà entendu parler du chemsex ? | - OUI  - NON |
| 7 | Vous estimez-vous capable de donner une définition au mot Chemsex ? | - OUI  - NON |
| 8 | Comment avez-vous eu connaissance de ces pratiques ? | - Formation initiale ou Formation continue  - Via la presse ou littérature ou internet  - Personnellement  - Autre : [Champ texte libre] |
| 9 | Selon vous, en termes d'épidémiologie : [Cochez la ou les propositions qui vous semblent juste] | - Entre 2021 et 2023, plus de 500 cas de complications liées au chemsex ont été signalées aux Centres d’Evaluation et d’Information sur la Pharmacodépendance-Addictovigilance (CEIP-A) en France  - Nous assistons à une baisse des complications infectieuses chez les usagers de chemsex  - En France, on estimerait le nombre de personnes potentiellement concernées par le chemsex entre 100 000 et 200 000  - L'enquête Décès en Relation avec l’Abus de Médicaments Et de Substances (DRAMES) menée par les Centres d’Evaluation et d’Information sur la Pharmacodépendance-Addictovigilance (CEIP-A) en France répertorie plus de 50 décès entre 2021 et 2022 liés au Chemsex  - 90% des cas de décès sont liés directement à des intoxications aux Cathinones de synthèse (3-MMC, 4-MMC...etc) |
| 10 | Selon vous, concernant les personnes concernées par le chemsex : [Cochez la ou les propositions qui vous semblent juste] | - Le chemsex touche indifféremment la population générale peu importe le sexe ou l’orientation sexuelle  - Le chemsex touche davantage les Hommes ayant des relations Sexuelles avec les Hommes (HSH)  - Le chemsex peut toucher davantage les usagers de PrEP (Prophylaxie Pré-Exposition au VIH, Emtricitabine / Ténofovir)  - Le chemsex peut également concerner des personnes non HSH |
| 11 | Cochez les molécules que vous pensez être utilisées fréquemment dans le chemsex : | - Cannabis/LSD/Champignons hallucinogènes  - GHB/GBL/Kétamine  - La Cocaïne, les Amphétamines et les Cathinones de synthèse : 3MMC, 4 MMC |
| 12 | Cochez les modes de consommation de drogues que vous connaissez : | - Sniffer  - Gober  - Slam  - Plug ou booty-bumping |
| 13 | Selon vous, quels sont les médicaments listés (sur prescription) pouvant être utilisés dans le cadre du chemsex ? | [Champ texte libre] |
| 14 | Vous estimez-vous capable de gérer des interactions entre certaines molécules utilisées dans le cadre du chemsex et certains médicaments ? | - Incapable  - Plutôt incapable  - Je ne sais pas  - Plutôt capable  - Tout à fait capable |
| 15 | Pouvez-vous en citer une ? | [Champ texte libre] |
| 16 | Connaissez-vous une base de données pour ces interactions ? | - OUI  - NON |
| 17 | Si oui, laquelle ? | [Champ texte libre] |
| 18 | Selon vous, la pratique du chemsex peut avoir des répercussions sur l'adhésion thérapeutique auprès de certains patients sur leurs traitements ? | - OUI  - NON |
| 19 | Seriez-vous en mesure d’identifier des signes de vulnérabilité ou des impacts de l’addiction (impacts sur la vie sociale, professionnelle, ou encore la vie sexuelle) suggérant une exposition au chemsex chez un patient ? | - Incapable  - Plutôt incapable  - Je ne sais pas  - Plutôt capable  - Tout à fait capable |
| Définition apparaissant afin que les participants n’ayant pas connaissance du sujet puissent répondre à la suite du questionnaire | ***Le chemsex se définit par l’usage de substances psychoactives (Cathinones de synthese : 3-MMC, 4-MMC, Cocaine, Methamphetamines…), avant ou pendant les rapports sexuels dans un but d’améliorer les performances, la durée et le plaisir sexuel avec des conséquences (sanitaires) individuelles (addictives et infectieuses) et populationnelles importantes.*** | |
| 20 | Dans votre exercice, avez-vous déjà eu des demandes de conseil ou d’orientation en lien avec le chemsex ? | - OUI  - NON  - NE SAIS PAS |
| 21 | Auriez-vous de l’appréhension à aborder la question du chemsex avec un patient que vous estimeriez à risque ? | - Aucune appréhension  - Plutôt pas d’appréhension  - Neutre  - Un peu d’appréhension  - Beaucoup d’appréhension |
| 22 | Si oui, pourquoi ? Quels sont les freins à la discussion ? | [Champ texte libre] |
| 23 | Comment vous positionnez-vous face à l’affirmation suivante : “Au comptoir, un patient chemsexeur est un patient à problèmes” ? | - Tout à fait d’accord  - Plutôt d’accord  - Ni en désaccord ni d’accord  - Plutôt pas d’accord  - Pas du tout d’accord |
| 24 | Selon vous, les outils d’information sur le chemsex et d’orientation vers des professionnels formés à la problématique, à destination des pharmaciens et des préparateurs en officine sont-ils suffisants ? | - OUI  - NON  - NE SAIS PAS |
| 25 | Selon vous, quel est le niveau global d’information sur la prévention auprès des patients usagers de chemsex ? | - Très bon  - Plutôt bon  - Neutre  - Plutôt mauvais  - Très mauvais |
| 26 | Dans une démarche de promotion à la santé et de prévention du chemsex, pensez-vous qu’au-delà de l’information sur les risques, les dimensions liées au plaisir et au lien social sont assez abordées en officine ? | - Tout à fait d’accord  - Plutôt d’accord  - Ni en désaccord ni d’accord  - Plutôt pas d’accord  - Pas du tout d’accord |
| 27 | Selon vous, est-il de la responsabilité du pharmacien de savoir aborder le sujet du Chemsex avec le patient, dans un objectif de prévention global ? | - OUI  - NON  - NE SAIS PAS |
| 28 | Si Non, Pourquoi ? | [Champ texte libre] |
| 29 | Si le patient présente des risques d’addictions ou des signes de vulnérabilité, sauriez-vous l’orienter vers les structures appropriées ? | - Très improbable  - Plutôt improbable  - Ne sais pas  - Plutôt probable  - Tout à fait probable |
| 30 | Dans une approche plus globale de prévention auprès des usagers de chemsex, pensez-vous pouvoir accompagner le patient en matière de prophylaxie pré-exposition au VIH (PrEP : ténofovir/emtricitabine), de vaccination et de réduction des risques d’infection ? | - Très improbable  - Plutôt improbable  - Ne sais pas  - Plutôt probable  - Tout à fait probable |
| 31 | Selon vous, comment pourrait-on améliorer la prise en charge du patient usager de chemsex ? | [Champ texte libre] |
|  | *[Validation du questionnaire]* |  |
