# Supplementary material for: Knowledge, attitudes and practices regarding chemsex prevention among community pharmacy professionals in France: a cross-sectional study
Source: BMJ Open. 2026 Apr 17;16(4):e107760. doi: 10.1136/bmjopen-2025-107760 (PMC13110612; doi:10.1136/bmjopen-2025-107760)
Supplement: Supplementary file 5 [file bmjopen-2025-107760.reviewer_comments.pdf]

PEER REVIEW HISTORY

BMJ Open publishes all reviews undertaken for accepted manuscripts. Reviewers are asked to complete a checklist review form and are provided with free text boxes to elaborate on their assessment. These free text comments are reproduced below.

ARTICLE DETAILS

Title (Provisional)

Knowledge, Attitudes, and Practices Regarding Chemsex Prevention Among Community Pharmacy Professionals in France: A Cross-Sectional Study

Authors

LEBRAT, MATTHIEU; BOUASLA, HOUSSEM EDDINE; LOGEROT, SOPHIE; Carrouel, Florence

---

VERSION 1 - REVIEW

**Reviewer**	**1**
**Name**	**Di Gennaro, Francesco**
**Affiliation**	**University of Bari Aldo Moro, Department of Precision and Regenerative Medicine and Ionian Area - (DiMePRe-J)**
**Date**	**08-Aug-2025**
**COI**	**None**

The manuscript addresses an important topic with potential public health implications. However, substantial revisions are required before it can be considered for publication. My concerns relate primarily to methodological clarity, validity of data interpretation, and alignment of conclusions with presented evidence. There is also a need for more detailed contextualization, improved statistical reporting, and strengthening of the discussion to address alternative explanations and limitations. Without these improvements, the robustness and reproducibility of the study are compromised.

Below my suggestions:

Clarity of Study Objectives

The introduction lacks a clear, concise statement of the study’s primary and secondary objectives. While the background provides context, the rationale is not sufficiently linked to a specific knowledge gap the study is trying to fill. The authors should explicitly state the research question and hypotheses.

Study Design Justification

The methodology section needs stronger justification for the chosen study design. It is not clear why this approach was selected over alternative designs (e.g., prospective cohort, matched controls), nor is there discussion on how potential biases inherent to the design were mitigated.

Sampling Strategy and Representativeness

The manuscript does not adequately describe the sampling frame, recruitment methods, or inclusion/exclusion criteria. Without this information, it is difficult to assess whether the sample is representative of the target population and whether selection bias may have occurred.

Measurement Validity and Reliability

The tools and procedures used for data collection require more detail, particularly regarding their validation in the study population. If adapted from previous instruments, the authors should provide references and describe any cultural or linguistic adaptation and pilot testing.

Statistical Analysis Transparency

The statistical methods are insufficiently detailed. It is unclear how missing data were handled, whether assumptions for statistical tests were checked, and whether adjustments for potential confounders were applied consistently. The authors should also justify their choice of significance threshold and provide confidence intervals for all key estimates.

Interpretation of Findings

Several conclusions in the results and discussion appear overstated relative to the data presented. The authors should avoid causal language in a cross-sectional/observational context and temper claims to reflect associations rather than cause–effect relationships.

Consideration of Confounding and Bias

The discussion does not adequately address potential confounders (e.g., socio-economic status, comorbidities, environmental exposures) that could explain the observed associations. Similarly, sources of bias—such as recall bias or measurement error—are not sufficiently discussed.

Public Health and Policy Relevance

While the topic has clear implications for public health, the authors should provide a more evidence-based and realistic assessment of how their findings could influence policy or practice. Recommendations currently appear too general and not directly supported by study results.

Limitations Section Strengthening

The limitations section is brief and does not critically reflect on the methodological weaknesses, generalizability issues, and potential misclassification. A transparent discussion of these points will enhance the credibility of the work.

**Reviewer**	**2**
**Name**	**Lee, Krystal**
**Affiliation**	**The University of Hong Kong, Psychiatry**
**Date**	**16-Dec-2025**

**COI**	**None**

1. Introduction - an important gap exists as to why and how community pharmacists can improve on the chemsex prevention in France, thus the importance of their knowledge/attitude?

2. Eligibility is not clearly explained, if all of the 3 criteria are required at the same time

3. No explanation on assumption of sample size calculation

4. No detail is given as to how the items in the survey were developed, esp if it was mentioned as the first of its kind, and what are the characteristics of the members of the expert committee, and for the two references quoted - all were not related to specially chemsex

5. Need to explain what is cognitive defriefing in line 153

6. Need to explain waht is GDPR (line 169)

7. A qualitative inquiry was included in the methods section but was not reported not in the result section

8. For table 4, most respondents reported inadequate prevention information to chemsex users, and information on referral tools, but high ability to refer chemsex users for service and support them with PrEP. -> seems contradictory.

9. One of the limitations is moderate variance in the measurements and identified unmeasured factors -> so what does it imply?

**Reviewer**	**3**
**Name**	**Khan, Mohammad Niaz Morshed**
**Affiliation**	**International Centre for Diarrhoeal Disease Research Bangladesh, Infectious Disease Division**
**Date**	**18-Dec-2025**
**COI**	**None**

This manuscript addresses a timely and important public health issue in France: the potential role of community pharmacies in chemsex prevention and harm reduction. Community pharmacists occupy a strategic position within primary care and may serve as an accessible point of contact for individuals using psychoactive substances in sexual contexts, giving this study clear relevance for practice and policy.

However, the manuscript requires substantial revision before it can be considered for publication. Several methodological and statistical issues limit the interpretability of the findings, and the epidemiological context relies largely on older prevalence data despite recent shifts in the chemsex landscape. In addition, the manuscript describes a mixed-methods design, including qualitative content analysis, yet the Results section presents only quantitative findings. This discrepancy between the stated methodology and the reported results is a major

issue that must be resolved, either by presenting the qualitative findings or by revising the methodological framing accordingly

1. Title & Abstract

The Abstract and Methods mention collecting qualitative data and performing content analysis. However, the Results section contains no qualitative themes, quotes, or content analysis. If qualitative data was not analyzed, all mentions of it must be removed. If it was analyzed, it must be presented to justify the "mixed methods" claim.

2. Introduction

1. The definition risks becoming overly inclusive. Listing alcohol, poppers, and PDE-5 inhibitors alongside cathinones, methamphetamine, and GHB/GBL may unintentionally blur the distinction between core chemsex practices driving the specific public health harms and substances that are often adjunct or co-consumed. Greater conceptual clarity would help avoid pathologizing lower-risk sexual practices and improve the paper’s utility for pharmacy professionals.

2. In France, community pharmacies already serve as a key access point for harm reduction tools, such as sterile injection kits (e.g., Stéribox) and dosing syringes. However, these interactions are largely transactional, with limited accompanying counseling or prevention messaging. Explicitly acknowledging this existing role would strengthen the rationale for engaging pharmacists more actively in chemsex prevention.

3. The introduction would benefit from clarifying whether the KAP framework employed is based on a validated or previously used instrument, particularly given the risk of social desirability bias when assessing attitudes and practices related to stigmatized behaviors.

4. While the introduction appropriately situates chemsex within MSM communities, it would be helpful to explicitly justify how this framing translates into the professional scope of community pharmacists, particularly given the potential for assumptions about sexual orientation in pharmacy encounters.

3. Methodology & Statistics

1. The construction of composite Knowledge, Attitude, and Practice scores requires demonstration of internal consistency. Reporting Cronbach’s alpha for each subscale would strengthen confidence that the items measure coherent constructs.

2. Recruitment via academic training supervisors may preferentially reach pharmacists who are more engaged with continuing education and university networks. As such, the sample may underrepresent more isolated practitioners, potentially limiting generalizability. Additionally, although the study is framed at the regional level, recruitment pathways appear primarily linked to Grenoble-based academic networks, which should be explicitly acknowledged as a limitation.

3. The use of forward stepwise regression, while common, has known limitations including coefficient instability and model overfitting. A theory-driven modeling strategy (e.g., hierarchical or full-model regression) may be more appropriate given the study’s applied public health aims. If stepwise selection is retained, stronger justification and sensitivity analyses would be helpful.

4. The power calculation is briefly mentioned, but key parameters (assumed effect size, alpha level, and primary outcome) are not specified. Providing these details would improve transparency and reproducibility.

5. The description of qualitative content analysis is brief. Additional detail regarding the analytic approach (e.g., inductive vs deductive coding, use of a coding framework, and strategies to enhance credibility) would strengthen methodological rigor.

4. Results

1. The term "practice score" may be misleading, as the items appear to assess perceived adequacy of resources and perceived needs rather than actual behaviors. Consider renaming to reflect the construct (e.g., 'perceived resource adequacy/need').

2. A substantial proportion incorrectly endorsed cannabis/hallucinogens as chemsex-related substances, suggesting a broad "drug use during sex" interpretation rather than the more specific set of substances commonly emphasized in chemsex harm profiles. This reinforces the need for clear definitional training.

3. Male gender was associated with higher knowledge scores. The paper should avoid essentialist explanations and consider alternative mechanisms (differential exposure to information, confidence in self-assessment, workplace role differences).

4. Given the stated inclusion of open-ended items and content analysis, the Results should report qualitative themes and provide representative quotes (with anonymized identifiers). If qualitative findings are minimal, the manuscript should clarify the role of open-ended data and avoid overstating mixed-methods contributions.

5. Discussion

1. The Discussion should explicitly highlight the low recognition of injection ("slam") as part of chemsex (18.1%). Given the elevated acute harms and blood-borne infection risks associated with injection, this knowledge gap may limit pharmacists' ability to provide harm-reduction counseling when dispensing sterile equipment.

2. The Discussion should address the apparent discrepancy between low demonstrated knowledge of drug–drug interactions/reference resources and relatively high self-reported confidence in referral capacity. This may indicate that professionals conceptualize chemsex primarily as a psychosocial issue requiring referral, potentially overlooking medication-safety responsibilities at the point of dispensing. Training could therefore integrate both referral pathways and practical medication-safety decision support.

3. Recruitment through academic training-supervisor networks and snowballing may have overrepresented pharmacists connected to university settings and continuing education. This may limit generalizability and could mean observed knowledge gaps represent a conservative estimate relative to the broader community pharmacy workforce.

4. The models explain a modest proportion of variance for attitude and perceived needs, suggesting that unmeasured factors (e.g., stigma-related beliefs, professional norms, prior training experiences) may be influential. This should be acknowledged as a limitation.

Recommendation: This paper requires Major Revisions to validate the statistical scores, correct the "mixed methods" discrepancy, and update the epidemiological context. The data is valuable, but the rigorous reporting is currently lacking.

VERSION 1 - AUTHOR RESPONSE

Point by point response to Reviewer 1:

Regarding the comment 1: “**The manuscript addresses an important topic with potential public health implications. However, substantial revisions are required before it can be considered for publication. My concerns relate primarily to methodological clarity, validity of data interpretation, and alignment of conclusions with presented evidence. There is also a need for more detailed contextualization, improved statistical reporting, and strengthening of the discussion to address alternative explanations and limitations. Without these improvements, the robustness and reproducibility of the study are compromised.”**

Author’s response: We thank Reviewer 1 for their constructive and detailed feedback. We acknowledge the concerns raised regarding methodological clarity, data interpretation, and alignment between the conclusions and the presented evidence. In response, we have substantially revised the manuscript, with particular improvements to the Introduction, Methods, and Discussion/Conclusions sections. These revisions include clearer description of the study design and analytical approach, improved statistical reporting, and expanded discussion of alternative interpretations and study limitations. Detailed, point-by-point responses to each specific comment are provided below.

Regarding the comment 2: “**Clarity of Study Objectives**
The introduction lacks a clear, concise statement of the study’s primary and secondary objectives. While the background provides context, the rationale is not sufficiently linked to a specific knowledge gap the study is trying to fill. The authors should explicitly state the research question and hypotheses.”

Author’s response: We thank the reviewer for this comment. This study was designed as an exploratory cross-sectional KAP study, and as such did not test a priori hypotheses. However, we agree that explicitly stating the research questions improves clarity. We have therefore revised the Introduction to clearly formulate the primary and secondary research questions and to specify the exploratory nature of the analyses (see Introduction, page 7, lines 159-162):

“Therefore, the primary objective of this study was to assess the current level of knowledge, attitudes, and practices (KAP) of community pharmacy health professionals regarding chemsex prevention in the Auvergne-Rhône-Alpes region of France. The secondary objective was to identify determinants associated with these KAP dimensions.”

We further developed a link between the rationale and the specific knowledge gap the study is trying to fill, page 7 lines 149-158:

“In order to develop strategies mobilizing pharmacists that could combine information provision, harm reduction strategy, effective screening and referral, it is first necessary to generate evidence-based data to inform prevention materials and guidelines.

A Knowledge–Attitude–Practice (KAP) study is particularly relevant in this context, as its exploratory approach allows to reveal what a target population knows, believes, and how they behave related to a health issue, thereby identifying gaps, misconceptions, and informational needs. With KAP baseline data, prevention tools and policies can be tailored to actual deficits and attitudes with their determinants rather than assumed needs. To date, no chemsex-specific KAP framework has been applied in the pharmacy field, making this exploratory work original."

Regarding the comment 3: "**Study Design Justification**
The methodology section needs stronger justification for the chosen study design. It is not clear why this approach was selected over alternative designs (e.g., prospective cohort, matched controls), nor is there discussion on how potential biases inherent to the design were mitigated."

Author's response: We thank the reviewer for this comment. We have revised the Study Design section to provide a clearer justification for the use of a cross-sectional KAP survey, explicitly positioning it as an exploratory and formative approach appropriate in a context where baseline data are scarce. We now discuss why alternative designs (prospective cohort, matched controls, interventional designs) were not suitable at this stage and have added a dedicated paragraph acknowledging potential biases inherent to cross-sectional surveys and describing the measures implemented to mitigate them. These revisions are intended to clarify the methodological rationale and strengthen the transparency of the study design. (pages 8-9, lines 167–188):

"A cross-sectional KAP survey was conducted to explore community pharmacy professionals' KAP regarding chemsex prevention. This design was selected because the study objective was exploratory and descriptive, aiming to generate baseline evidence in an area where empirical data are scarce. KAP surveys are commonly used in public health research as a formative approach to identify knowledge gaps, prevailing attitudes, and current practices prior to the development of targeted prevention or educational interventions. Alternative study designs, such as prospective cohort studies or matched control designs, were deemed less appropriate at this stage. Indeed, such designs require predefined exposure–outcome relationships or temporal hypotheses, which were not feasible given the absence of prior data on pharmacists' involvement in chemsex prevention. Similarly, interventional designs would have been premature without an initial assessment of baseline needs and contextual factors.
To mitigate potential biases inherent to cross-sectional survey designs, several measures were implemented. Participation was voluntary and anonymous to reduce social desirability bias, and the questionnaire employed neutral, non-judgmental wording. Broad dissemination aimed to limit selection bias, and the study adhered to the Checklist for Reporting Of Survey Studies (CROSS) to enhance transparency and methodological rigor [14] (see Supplementary material S1). While the cross-sectional nature of the study precludes causal inference and temporal assessment, it is well suited to its intended purpose as a preliminary needs assessment informing future intervention design.
This study was conducted between February and March 2025."

Regarding the comment 4: "**Sampling Strategy and Representativeness**
The manuscript does not adequately describe the sampling frame, recruitment methods, or inclusion/exclusion criteria. Without this information, it is difficult to

assess whether the sample is representative of the target population and whether selection bias may have occurred.”

Author’s response: We thank the reviewer for this comment. We have revised the Methods section to explicitly describe the sampling frame, recruitment procedures, and inclusion/exclusion criteria (page 9, lines 192–208). The sampling strategy is now clearly identified as non-probability sampling (convenience and snowball sampling), and we have clarified that the sample is not statistically representative of the target population. Although the sample is not statistically representative, it shares some demographic characteristics with the national pharmacy workforce. A limitation addressing potential selection bias and limited generalizability has been added to the Discussion section (pages 37-38, lines 629–641). The limitation has also been highlighted in the bullet points after the abstract. (page 3, lines 65–73)

“Study population, sampling strategy, recruitment, and sample size

This study used a non-probability sampling strategy combining convenience and snowball sampling. The initial sampling frame consisted of community pharmacy training supervisors affiliated with the pharmacy faculties of Grenoble and Lyon, who were contacted via a mailing list. These supervisors were invited to participate and to disseminate the study invitation to colleagues working in community pharmacies. Additional participants were recruited through direct contact and professional networks (word of mouth), further contributing to a convenience-based recruitment approach.
Inclusion criteria were all of the following: (i) adults aged ≥18 years, (ii) pharmacists, pharmacy technicians, or students in training, and (iii) currently working in a community pharmacy located in the Auvergne-Rhône-Alpes region of France. Participants meeting all inclusion criteria were eligible for participation. Individuals who did not provide informed consent were excluded.
A minimum sample size of at least 196 participants was targeted to allow estimation of proportions with maximum variance (p = 0.50) with a two-sided 95% confidence interval and a margin of error of ±7% (chosen as a pragmatic compromise between acceptable precision and feasibility). The calculation was performed using the pwr package in R (version 4.2.1).”

“Potential selection bias exists with the use of convenience and snowball sampling. Participants with a particular interest in sexual health or harm reduction may have been more likely to respond, as well as those more engaged with continuing education or university networks, since the initial sampling frame was derived from pharmacy training supervisors affiliated with the faculties of Grenoble and Lyon, which may have led to an overrepresentation of professionals connected to these academic networks. Consequently, observed knowledge gaps may represent a conservative estimate relative to the broader community pharmacy workforce with findings that may not be fully generalizable to all community pharmacy professionals in the Auvergne-Rhône-Alpes region or elsewhere in France. However, cautious interpretation is warranted, as the sample shares several characteristics with the national pharmacy workforce (including a predominance of female pharmacists [24]) and includes professionals from diverse professional backgrounds and age groups.”

“• Non-probability sampling, which may limit representativeness and generalizability.”

Regarding the comment 5: **“Measurement Validity and Reliability The tools and procedures used for data collection require more detail, particularly regarding their validation in the study population. If adapted from previous instruments, the authors should provide references and describe any cultural or linguistic adaptation and pilot testing.”**

Author’s response: We thank the reviewer for this comment. We have substantially revised the “Questionnaire design and validation” section to provide additional detail on the development and validation procedures. We now explicitly state that the questionnaire was newly developed for this study, describe the theoretical frameworks guiding item construction, and report the procedures used to assess face and content validity through an expert committee.

We have also clarified the cultural and linguistic context, specifying that the questionnaire was developed in French for a French population, and described the cognitive debriefing process conducted with community pharmacy professionals and students to ensure clarity, comprehension, and contextual relevance.

In addition, internal consistency of the composite Knowledge, Attitude, and Perceived resource adequacy/need scores was assessed using Cronbach’s alpha based on the study sample, yielding acceptable to good values ($\alpha = 0.77$, 0.61, and 0.81, respectively). These analyses were added to improve transparency while maintaining the exploratory nature of the instrument (pages 10–11, lines 210–246).

“Questionnaire design and validation

The questionnaire to analyze chemsex prevention KAP was newly developed for this study. As no validated chemsex-specific KAP questionnaire exists to date, general methodological guidelines for KAP survey development and validation were used. It was designed following a literature review conducted by M.L. and H.B., in accordance with the World Health Organization’s recommendations for KAP survey design [25] and the methodological framework proposed by Andrade et al.’s [23]. Item generation was informed by the reviewed literature, French public health report on chemsex [7], and discussions within the research team. An initial pool of 48 items was generated.
Face and content validity were assessed by an Expert Committee who independently reviewed items using standardized rating sheets. The Expert Committee consisted of 3 pharmacists with experience in community practice and health prevention, and 2 physicians specialized in addiction medicine and/or mental health, all familiar with chemsex-related issues through clinical or public health practice. Items rated as unsatisfactory were revised or removed, and the final version comprising 31 items was approved by the committee.
As the questionnaire was originally developed in French for a French population, no linguistic translation was required. Cultural relevance and comprehension were addressed through qualitative pretesting.
Cognitive debriefing interviews were conducted with four pharmacists and two final-year pharmacy students to refine clarity, comprehension, and relevance of items. During individual interviews, participants were asked to explain their understanding of each item, the meaning attributed to key terms, and the rationale for their responses, in line with recommended best practices for questionnaire pretesting [26]. Feedback from these interviews was used to refine item wording and ensure cultural and contextual appropriateness.

Formal psychometric validation beyond assessment of internal consistency (e.g., test–retest reliability or construct validation) was not performed, as the primary objective of this study was exploratory and descriptive.
The final validated questionnaire was hosted on LimeSurvey®, accessible online, and required about 5-10 minutes to complete (S2 Table).
It included 31 questions (25 closed, 6 open-ended), divided into four sections:
(i) Sociodemographic data (5 questions),
(ii) Chemsex knowledge (14),
(iii) Attitudes toward prevention (6)
(iv) Practices and perceived resource adequacy / needs (6)."

Regarding the comment 6: **"Statistical Analysis Transparency**
The statistical methods are insufficiently detailed. It is unclear how missing data were handled, whether assumptions for statistical tests were checked, and whether adjustments for potential confounders were applied consistently. The authors should also justify their choice of significance threshold and provide confidence intervals for all key estimates."

Author's response: We thank the reviewer for this comment. The "Data analysis" section has been substantially revised to improve transparency and clarity. We now explicitly describe the handling of missing data, specifying the use of complete-case analyses and the rationale for assuming missingness at random.

We have also clarified the construction of composite scores, detailed the covariate selection strategy for multivariable models, and stated that regression assumptions were assessed using standard diagnostic procedures. The reporting of regression results has been clarified to confirm that regression coefficients, 95% confidence intervals, and p-values were provided for all key estimates. Finally, we have justified the use of a two-sided significance threshold of α = 0.05 in line with conventional practice in exploratory public health research (pages 12-14, lines 265–317):

"Data analysis

Descriptive statistics were used to summarize participant characteristics and questionnaire responses. Descriptive statistics (percentages) were used for closed-ended items, with means and standard deviations reported for score variables. All received questionnaires were included in descriptive analysis. For inferential analyses, only complete questionnaires for the relevant variables were included (complete-case analysis). Missing data were not imputed. Missingness primarily resulted from participants discontinuing the questionnaire before completion, as suggested by the pattern of item completion in the received questionnaires, and was therefore assumed to be Missing At Random (MAR).
New composite score variables were created:
Knowledge score: Sum of items 6, 7, 9–19 (1 point per correct or valid open-ended response; Likert items scored 0–3). The theoretical score ranged from 0 to 29 points.
Attitude score toward chemsex prevention: Sum of items 21, 23, 26, and 27 (Likert items scored 0–4; specific scoring for item 27 based on agreement with the pharmacists' role in prevention). The theoretical score ranged from 0 to 13 points.
Perceived resource adequacy/need score (capturing the perceived adequacy of available resources and practical ability to refer and support patients): Sum of items 24, 25, 29, and

30 (Likert items scored 0–4; specific scoring for item 24 based on perceived adequacy of available resources). The theoretical score ranged from 0 to 13 points.
These composite scores were constructed for descriptive and exploratory purposes. Internal consistency was assessed using Cronbach's alpha; however, the questionnaire was not intended as a fully psychometrically validated scale.

Qualitative free-text responses underwent conventional content analysis [27]. An inductive approach was applied, allowing categories to emerge directly from the data without a predefined coding framework. Coding was conducted iteratively through familiarization, open coding, and category development, and continued until category saturation was achieved. Meaning units were identified and grouped into categories; multiple categories could be assigned to a single response. Two researchers (M.L. and H.B.) independently coded all responses, with discrepancies resolved through discussion and consensus. Coding decisions were documented to ensure analytic transparency, and representative quotes were selected to illustrate categories. Descriptive frequencies of categories were calculated to identify prominent patterns in the data.
To identify determinants associated with knowledge, attitude, and practice/ perceived resource adequacy/need scores, separate multivariable linear regression models were fitted for each outcome. Covariates were selected a priori based on theoretical relevance and previous literature and included sociodemographic characteristics (items 1, 2, 4, 5: profession, pharmacy typology, gender, and age), source of awareness about chemsex (item 8), history of chemsex-related advice requests (item 20), and KAP scores.
Years of practice (item 3) were excluded to avoid collinearity with age.
Forward stepwise selection based on the Akaike Information Criterion (AIC) was then applied as an exploratory variable reduction strategy within this predefined set of covariates. Assumptions of linear regression (linearity, normality of residuals, homoscedasticity, and absence of multicollinearity) were assessed using residual plots.
Regression coefficients (β), 95% confidence intervals (CI), and p-values were reported for all models. A two-sided significance threshold of $\alpha = 0.05$ was used, consistent with conventional practice in exploratory public health research. Given the exploratory nature of the study, the regression analyses were intended to identify potential associations rather than establish causal relationships.
Statistical analyses were performed using R (software version 4.2.1; R Core Team, 2022)."

Regarding the comment 7: **"Interpretation of Findings**
Several conclusions in the results and discussion appear overstated relative to the data presented. The authors should avoid causal language in a cross-sectional/observational context and temper claims to reflect associations rather than cause–effect relationships."

<u>Author's response</u>: We thank the reviewer for this important comment. We carefully revised the Results and Discussion sections to avoid causal language and overinterpretation of findings, in line with the cross-sectional and exploratory design of the study.

Specifically, we replaced causal terminology (e.g., "predictors") with neutral association-based wording (e.g., "factors associated with"). We systematically rephrased explanatory statements in the Discussion to clarify that observed relationships represent associations rather than cause–effect relationships, and we explicitly acknowledged the inability to infer directionality.

In addition, we tempered interpretations by qualifying findings as specific to the studied population and by adding cautionary statements where speculative mechanisms were previously implied. These revisions were made throughout the Results and Discussion sections to ensure that conclusions accurately reflect the observational nature of the data.

Regarding the comment 8: **“Consideration of Confounding and Bias**
The discussion does not adequately address potential confounders (e.g., socio-economic status, comorbidities, environmental exposures) that could explain the observed associations. Similarly, sources of bias—such as recall bias or measurement error—are not sufficiently discussed.”

Author’s response: We thank the reviewer for highlighting the importance of confounding and bias. We have expanded the Strengths and limitations section to explicitly discuss potential unmeasured confounders (e.g., prior training, exposure to key populations, socioeconomic context of practice) that could influence the observed associations. We also added a detailed discussion of potential sources of bias, including selection bias, recall bias, social desirability bias, and measurement error related to self-reported data and knowledge score construction. These additions clarify the limitations of the study and reinforce the need for cautious interpretation of the findings. (pages 37-39, lines 628–671):

“The cross-sectional design prevents causal conclusions. Potential selection bias exists with the use of convenience and snowball sampling. Participants with a particular interest in sexual health or harm reduction may have been more likely to respond, as well as those more engaged with continuing education or university networks, since the initial sampling frame was derived from pharmacy training supervisors affiliated with the faculties of Grenoble and Lyon, which may have led to an overrepresentation of professionals connected to these academic networks. Consequently, observed knowledge gaps may represent a conservative estimate relative to the broader community pharmacy workforce with findings that may not be fully generalizable to all community pharmacy professionals in the Auvergne-Rhône-Alpes region or elsewhere in France. However, cautious interpretation is warranted, as the sample shares several characteristics with the national pharmacy workforce (including a predominance of female pharmacists [38]) and includes professionals from diverse professional backgrounds and age groups.
Additional sources of bias and confounding should also be considered. Variables such as prior training in sexual health or addiction, workload, pharmacy size, socioeconomic characteristics of the catchment area, and frequency of exposure to key populations (e.g., MSM or PrEP users) were not measured and may have influenced both knowledge and attitudes toward chemsex prevention. Consequently, residual confounding cannot be excluded. Information bias may have occurred due to self-reported data, including recall bias regarding prior chemsex-related requests and social desirability bias, particularly for attitudes and perceived practices related to stigmatized behaviors. Participants may have overreported favorable attitudes toward prevention or perceived preparedness. To mitigate this risk, the questionnaire was anonymous, self-administered, and completed online, with no identifying information collected. Despite face and content validation and cognitive debriefing during questionnaire development, some measurement error related to item framing or response scales cannot be entirely excluded.
The multivariable regression models explained a moderate variance for knowledge (35.8%) but less for attitudes (11%) and perceived resource adequacy/need (8%). This suggests that while individual characteristics and prior exposure to chemsex-related situations partly explain knowledge levels, attitudes and perceived practices are likely shaped by additional unmeasured factors. These may include personal beliefs, perceived

professional role boundaries, prior trainings, moral or cultural representations of sexuality and substance use, prior informal experiences with chemsex-related situations, organizational context, and perceived institutional support. Moreover, the use of forward stepwise regression may have introduced model instability or overfitting, and the identified associations should therefore be interpreted as exploratory and hypothesis-generating rather than confirmatory.
Finally, although internal consistency of the composite scores was acceptable, the questionnaire was newly developed and not intended as a fully psychometrically validated instrument. Further research should aim to confirm these findings and to conduct comprehensive psychometric validation of chemsex-specific KAP tools for pharmacy professionals."

Regarding the comment 9: **"Public Health and Policy Relevance**
While the topic has clear implications for public health, the authors should provide a more evidence-based and realistic assessment of how their findings could influence policy or practice. Recommendations currently appear too general and not directly supported by study results."

Author's response: We thank the reviewer for this constructive comment, which helped us strengthen the Discussion section.
In response, we revised the Discussion to more explicitly link our policy and practice implications to the study's empirical findings. We now ground recommendations in specific results, including: (i) the heterogeneous and often limited levels of chemsex-related knowledge among community pharmacy professionals; (ii) the positive association between knowledge and attitudes toward prevention; (iii) the very low proportion of professionals with direct experience providing chemsex-related advice; and (iv) the widespread perception of insufficient tools and resources.
We also clarified how these findings translate into realistic and actionable implications for practice, particularly regarding targeted education and training for community pharmacy staff and the need for non-judgmental, patient-centered prevention approaches. These implications are discussed within the existing scope of pharmacists' public health missions (addiction prevention, harm reduction, sexual health services), thereby reinforcing their feasibility and policy relevance.
Finally, we explicitly acknowledge the limits of causal inference inherent to the cross-sectional design and avoid overgeneralization, in order to provide a balanced and evidence-based assessment of how our findings may inform future public health strategies. (Pages 32-37)

Regarding the comment 10: **"Limitations Section Strengthening**
The limitations section is brief and does not critically reflect on the methodological weaknesses, generalizability issues, and potential misclassification. A transparent discussion of these points will enhance the credibility of the work."

Author's response: We thank the reviewer for this important comment. We have substantially revised and expanded the Strengths and limitations section to provide a more transparent and critical discussion of the study's methodological constraints. Specifically, we now explicitly address the limitations related to the cross-sectional design and the resulting inability to infer causality, potential selection bias associated with convenience and snowball sampling, and the limited generalizability of the findings beyond the study region.

In addition, we discuss potential sources of confounding, including unmeasured factors such as prior training in sexual health or addiction, pharmacy characteristics, and population profiles, as well as information biases related to self-reported data, recall bias, social desirability bias, and possible measurement limitations of the questionnaire. We also report the explanatory power of the regression models to acknowledge the influence of unmeasured determinants (pages 37-39, lines 618–671).

Point by point response to Reviewer 2:

Regarding the comment 1: **"Introduction - an important gap exists as to why and how community pharmacists can improve on the chemsex prevention in France, thus the importance of their knowledge/attitude?"**

Author's response: We thank the reviewer for this insightful comment. We have revised the Introduction to clarify both why community pharmacy professionals are relevant actors in chemsex prevention and why a KAP approach (knowledge, attitudes, and practices) is appropriate. Specifically, we now highlight (i) the strategic role of frontline professionals in prevention and early intervention, (ii) the pharmacists' legal obligation under French law to contribute to addiction prevention, and (iii) the current absence of published data on community pharmacists' involvement in chemsex prevention. We further explain that, in the absence of baseline data, a KAP study is an appropriate exploratory method to identify knowledge gaps, attitudes, and practices in order to inform future prevention tools and policies (pages 6–7, lines 126–162):

"However, community pharmacies, despite their high accessibility and potential for frontline health promotion, remain largely uninvolved in structured chemsex prevention efforts with no related published data.

Under French law, pharmacists have a legal obligation to contribute to the prevention of drug addiction, as defined in the Public Health Code [22]. In practice, community pharmacies already serve as a key access point for harm reduction through established interventions such as the dispensation of sterile injection kits (e.g., Stéribox) and other public health campaigns (e.g., tobacco-free month or Dry January).

Nevertheless, most interactions in pharmacies are still predominantly transactional, centered on product dispensing or brief public health messaging, with limited time and opportunity for in-depth counseling or tailored prevention discussions. Explicitly building on this existing role while moving beyond transactional encounters could strengthen pharmacists' contribution to chemsex prevention and harm reduction.

Mobilizing pharmacists could improve early detection of risky behaviors, facilitate brief interventions, and enhance referral pathways within a comprehensive and integrated prevention strategy. In routine pharmacy practice, pharmacists interact with patients primarily through medication dispensing, harm reduction tools, and requests for information, rather than through discussions of sexual identity. Their potential role in chemsex prevention therefore lies in responding to substance-related, sexual health–related, or medication-related cues (e.g., requests for HIV PrEP, post-exposure prophylaxis, STI treatment, or harm reduction equipment), irrespective of patients' sexual orientation. This behavior- and needs-based approach aligns with pharmacists' professional scope and ethical obligations, while avoiding assumptions about identity.

In order to develop strategies mobilizing pharmacists that could combine information provision, harm reduction strategy, effective screening and referral, it is first necessary to generate evidence-based data to inform prevention materials and guidelines. A

Knowledge–Attitude–Practice (KAP) study is particularly relevant in this context, as its exploratory approach allows to reveal what a target population knows, believes, and how they behave related to a health issue, thereby identifying gaps, misconceptions, and informational needs [23]. With KAP baseline data, prevention tools and policies can be tailored to actual deficits and attitudes with their determinants rather than assumed needs. To date, no chemsex-specific KAP framework has been applied in the pharmacy field, making this exploratory work original.

Therefore, the primary objective of this study was to assess the current level of knowledge, attitudes, and practices (KAP) of community pharmacy health professionals regarding chemsex prevention in the Auvergne-Rhône-Alpes region of France. The secondary objective was to identify determinants associated with these KAP dimensions."

Regarding the comment 2: **"Eligibility is not clearly explained, if all of the 3 criteria are required at the same time"**

Author's response: We thank the reviewer for this comment. We have clarified that all inclusion criteria had to be met simultaneously by adding the wording "all of the following" and the sentence "Participants meeting all inclusion criteria were eligible for participation." to the eligibility criteria in the Methods section (page 9, lines 199–203):

"Inclusion criteria were all of the following: (i) adults aged ≥18 years, (ii) pharmacists, pharmacy technicians, or students in training, and (iii) currently working in a community pharmacy located in the Auvergne-Rhône-Alpes region of France. Participants meeting all inclusion criteria were eligible for participation. Individuals who did not provide informed consent were excluded."

Regarding the comment 3: **"No explanation on assumption of sample size calculation"**

Author's response: We thank the reviewer for this comment. We have revised the Methods section to explicitly describe the assumptions underlying the sample size calculation, including the assumed proportion, significance level, and precision of the estimate, as well as the statistical framework used (page 9, lines 204–208).

"A minimum sample size of at least 196 participants was targeted to allow estimation of proportions with maximum variance (p = 0.50) with a two-sided 95% confidence interval and a margin of error of ±7% (chosen as a pragmatic compromise between acceptable precision and feasibility). The calculation was performed using the pwr package in R (version 4.2.1)."

Regarding the comment 4: **"No detail is given as to how the items in the survey were developed, esp if it was mentioned as the first of its kind, and what are the characteristics of the members of the expert committee, and for the two references quoted - all were not related to specially chemsex"**

Author's response: We thank the reviewer for this important comment. We have substantially revised the Methods section to detail the development of the questionnaire, including the item generation process, sources informing item content, and the steps undertaken to assess face and content validity. We have also clarified the composition and

expertise of the Expert Committee involved in the review process, as well as the qualitative pretesting procedures. Finally, we now explicitly state that, in the absence of validated chemsex-specific KAP instruments, established methodological guidelines for KAP survey design and validation were used (pages 10-11, lines 212–239).

"The questionnaire to analyze chemsex prevention KAP was newly developed for this study. As no validated chemsex-specific KAP questionnaire exists to date, general methodological guidelines for KAP survey development and validation were used. It was designed following a literature review conducted by M.L. and H.B., in accordance with the World Health Organization's recommendations for KAP survey design [25] and the methodological framework proposed by Andrade et al.'s [23]. Item generation was informed by the reviewed literature, French public health report on chemsex [7], and discussions within the research team. An initial pool of 48 items was generated.
Face and content validity were assessed by an Expert Committee who independently reviewed items using standardized rating sheets. The Expert Committee consisted of 3 pharmacists with experience in community practice and health prevention, and 2 physicians specialized in addiction medicine and/or mental health, all familiar with chemsex-related issues through clinical or public health practice. Items rated as unsatisfactory were revised or removed, and the final version comprising 31 items was approved by the committee.
As the questionnaire was originally developed in French for a French population, no linguistic translation was required. Cultural relevance and comprehension were addressed through qualitative pretesting.
Cognitive debriefing interviews were conducted with four pharmacists and two final-year pharmacy students to refine clarity, comprehension, and relevance of items. During individual interviews, participants were asked to explain their understanding of each item, the meaning attributed to key terms, and the rationale for their responses, in line with recommended best practices for questionnaire pretesting [26]. Feedback from these interviews was used to refine item wording and ensure cultural and contextual appropriateness.
Formal psychometric validation beyond assessment of internal consistency (e.g., test–retest reliability or construct validation) was not performed, as the primary objective of this study was exploratory and descriptive."

Regarding the comment 5: **"Need to explain what is cognitive debriefing in line 153"**

<u>Author's response</u>: We thank the reviewer for this comment. We have clarified the meaning and purpose of cognitive debriefing by adding a brief description of the method and an appropriate methodological reference in the Methods section to improve transparency (page 10-11, lines 230–236).

"Cognitive debriefing interviews were conducted with four pharmacists and two final-year pharmacy students to refine clarity, comprehension, and relevance of items. During individual interviews, participants were asked to explain their understanding of each item, the meaning attributed to key terms, and the rationale for their responses, in line with recommended best practices for questionnaire pretesting [26]. Feedback from these interviews was used to refine item wording and ensure cultural and contextual appropriateness."

Regarding the comment 6: **"Need to explain waht is GDPR (line 169)"**

Author's response: We thank the reviewer for this comment. We have clarified what the General Data Protection Regulation (GDPR) is and revised the Methods section to explicitly state how data protection requirements were addressed in this study (page 11, lines 250–256):

"Data were collected anonymously via LimeSurvey® (dedicated server with 2048-bit SSL data encryption) and exported to R for analysis. No IP addresses or other personally identifiable information were recorded, and age and similar variables were collected in grouped categories to prevent direct or indirect identification. The General Data Protection Regulation (GDPR), the European Union legal framework governing the protection of personal data, was taken into account. As only fully anonymized data were collected, the study complied with GDPR requirements."

Regarding the comment 7: "**A qualitative inquiry was included in the methods section but was not reported not in the result section**"

Author's response: We thank the reviewer for highlighting this inconsistency. We have revised the Methods section to clarify the qualitative analysis procedure (inductive content analysis) and have fully reported the qualitative findings in the Results section. Qualitative results are now presented through emergent categories supported by verbatim quotations, detailed in Tables 2, 3, and 4, and described in the accompanying text (page 13, lines 290-300, lines 359-365; page 17, lines 355-357; page 23, lines 386-400; page 24, lines 405-420; pages 28-29, lines 447-487; Tables 2–4).

Regarding the comment 8: "**For table 4, most respondents reported inadequate prevention information to chemsex users, and information on referral tools, but high ability to refer chemsex users for service and support them with PrEP. -> seems contradictory.**»

Author's response: We thank the reviewer for this insightful comment. We have revised the Discussion section to explicitly address this apparent contradiction. We now clarify that the reported ability to refer individuals likely reflects familiarity with general sexual health and addiction referral pathways, whereas the perceived lack of information and tools relates to the absence of chemsex-specific resources. Potential explanatory factors, including social desirability bias and experiential learning, are also discussed (pages 35-36, lines 573–595):

"At the same time, most participants perceived prevention efforts and available tools as insufficient, highlighting a lack of structured resources and guidance. Despite this, many reported feeling able to refer at-risk patients and expressed willingness to support related services such as HIV PrEP, vaccination, and STI risk reduction, indicating a foundation on which to build.
Although this contrast may appear contradictory, it likely reflects the distinction between general referral capacity and chemsex-specific preparedness. Referral to sexual health or addiction services often relies on existing, well-established pathways with which pharmacy professionals are already familiar, whereas chemsex-specific prevention requires tailored knowledge, tools, and training that are currently lacking. The discrepancy between low demonstrated knowledge of drug–drug interactions or reference resources and high self-reported referral capacity further suggests that professionals may conceptualize chemsex

primarily as a psychosocial issue requiring referral, potentially overlooking medication-safety responsibilities at the point of dispensing. Training initiatives could therefore benefit from integrating both referral pathways and practical medication-safety decision support. In addition, social desirability bias may have contributed to overreporting perceived referral capacity.
Interestingly, having previously received chemsex-related questions was negatively associated with the perceived adequacy of resources and needs ($p = 0.047$), which may reflect differences in perceived preparedness among professionals with prior exposure; however, reverse causality and unmeasured confounding are possible. Overall, these findings highlight the importance of targeted training and practical tools to bridge the gap between willingness to act and effective chemsex-specific prevention practices."

Regarding the comment 9: "**One of the limitations is moderate variance in the measurements and identified unmeasured factors -> so what does it imply?** »

<u>Author's response</u>: We thank the reviewer for this comment. We have expanded the limitations section to interpret the implications of the moderate and low explained variance observed in the regression models. We now clarify that attitudes and practices are likely influenced by additional unmeasured individual, contextual, and organizational factors, and we discuss how this limits causal interpretation while highlighting directions for future research (page 39, lines 656–666):

"The multivariable regression models explained a moderate variance for knowledge (35.8%) but less for attitudes (11%) and perceived resource adequacy/need (8%). This suggests that while individual characteristics and prior exposure to chemsex-related situations partly explain knowledge levels, attitudes and perceived practices are likely shaped by additional unmeasured factors. These may include personal beliefs, perceived professional role boundaries, prior trainings, moral or cultural representations of sexuality and substance use, prior informal experiences with chemsex-related situations, organizational context, and perceived institutional support. Moreover, the use of forward stepwise regression may have introduced model instability or overfitting, and the identified associations should therefore be interpreted as exploratory and hypothesis-generating rather than confirmatory."

Point by point response to Reviewer 3:

Regarding the comment 1: "**This manuscript addresses a timely and important public health issue in France: the potential role of community pharmacies in chemsex prevention and harm reduction. Community pharmacists occupy a strategic position within primary care and may serve as an accessible point of contact for individuals using psychoactive substances in sexual contexts, giving this study clear relevance for practice and policy.**
However, the manuscript requires substantial revision before it can be considered for publication. Several methodological and statistical issues limit the interpretability of the findings, and the epidemiological context relies largely on older prevalence data despite recent shifts in the chemsex landscape. In addition, the manuscript describes a mixed-methods design, including qualitative content analysis, yet the Results section presents only quantitative findings. This discrepancy between the stated methodology and the reported results is a major

issue that must be resolved, either by presenting the qualitative findings or by revising the methodological framing accordingly"

Author's response: We thank Reviewer 3 for this comprehensive and constructive assessment of our manuscript. In response, we have undertaken substantial revisions to address each of the issues raised.

First, we have revised the Methods, Results and Discussion sections to clarify the statistical analyses performed, to better justify the analytical choices, and to explicitly acknowledge the limitations related to the cross-sectional design and the absence of causal inference (pages 8-39).

Second, the Introduction has been updated to include more recent epidemiological data on chemsex in France and Europe, reflecting recent developments around chemsex with results from the last systematic review and meta-analysis from 2025 on the subject (pages 4–7).

Third, to resolve the inconsistency between the stated mixed-methods approach and the reported findings, we have expanded the description of the qualitative methodology and now fully report the qualitative results derived from open-ended questionnaire responses. These findings are presented in the Results section through inductive content analysis, with emergent categories and illustrative verbatim quotations (Tables 2–4, pages 15–31).

We believe these revisions substantially improve the methodological coherence, transparency, and interpretability of the study.

Regarding the comment 2: "**The Abstract and Methods mention collecting qualitative data and performing content analysis. However, the Results section contains no qualitative themes, quotes, or content analysis. If qualitative data was not analyzed, all mentions of it must be removed. If it was analyzed, it must be presented to justify the "mixed methods" claim.** »

Author's response: We thank the reviewer for this important comment. The qualitative data collected through open-ended questionnaire items were analyzed using inductive content analysis. In the revised manuscript, we now fully report these qualitative findings in the Results section through the presentation of emergent categories supported by illustrative verbatim quotations, thereby justifying the mixed-methods approach. The qualitative methodology has also been clarified in the Methods section to ensure consistency across sections (page 13, lines 290-300, lines 359-365; page 17, lines 355-357; page 23, lines 386-400; page 24, lines 405-420; pages 28-29, lines 447-487; Tables 2–4).

Regarding the comment 3: "**The definition risks becoming overly inclusive. Listing alcohol, poppers, and PDE-5 inhibitors alongside cathinones, methamphetamine, and GHB/GBL may unintentionally blur the distinction between core chemsex practices driving the specific public health harms and substances that are often adjunct or co-consumed. Greater conceptual clarity would help avoid pathologizing lower-risk sexual practices and improve the paper's utility for pharmacy professionals.**"

Author’s response: We thank the reviewer for this important and nuanced comment. We have revised the Introduction to improve conceptual clarity by explicitly distinguishing between substances that are central to chemsex practices and drive the associated public health harms (e.g., methamphetamine, cathinones, GHB/GBL) and substances that are more commonly adjunct or co-consumed in sexualized drug use contexts (e.g., alcohol, poppers, PDE-5 inhibitors). We now clarify that the latter do not define chemsex when used alone, in order to avoid over-inclusivity and unintended pathologization of lower-risk sexual practices, and to enhance the paper’s relevance for pharmacy professionals (page 4, lines 79–89):

“It primarily includes the use of potent psychoactive substances associated with a high risk of dependence, toxicity, and sexual health harms, notably methamphetamine, cathinones (mephedrone, 4-methylmethcathinone [4MMC], 3-methylmethcathinone [3MMC]), and gamma-hydroxybutyrate / gamma-butyrolactone (GHB/GBL).
Other substances like alcohol, ketamine, cocaine, poppers, and phosphodiesterase type 5 (PDE-5) inhibitors, may be used concomitantly in sexualized drug use contexts. However, these substances are generally considered adjunct or co-consumed substances and, when used alone, do not constitute chemsex. Importantly, the mere use of drugs during sex is not sufficient to define chemsex; rather, it is more accurately understood as a distinct behavioral and cultural phenomenon.”

Regarding the comment 4: “**In France, community pharmacies already serve as a key access point for harm reduction tools, such as sterile injection kits (e.g., Stéribox) and dosing syringes. However, these interactions are largely transactional, with limited accompanying counseling or prevention messaging. Explicitly acknowledging this existing role would strengthen the rationale for engaging pharmacists more actively in chemsex prevention.**”

Author’s response: We thank the reviewer for this insightful comment. We have revised the Introduction to explicitly acknowledge that community pharmacies in France already play a key role in harm reduction through established interventions such as the dispensation of sterile injection kits and public health prevention campaigns. We also clarify that these interactions are predominantly transactional, with limited accompanying counseling or prevention messaging. This clarification strengthens the rationale for exploring how pharmacists could move beyond existing roles to engage more actively in chemsex prevention (page 6, lines 129–138).

“Under French law, pharmacists have a legal obligation to contribute to the prevention of drug addiction, as defined in the Public Health Code [22]. In practice, community pharmacies already serve as a key access point for harm reduction through established interventions such as the dispensation of sterile injection kits (e.g., Stéribox) and other public health campaigns (e.g., tobacco-free month or Dry January).
Nevertheless, most interactions in pharmacies are still predominantly transactional, centered on product dispensing or brief public health messaging, with limited time and opportunity for in-depth counseling or tailored prevention discussions. Explicitly building on this existing role while moving beyond transactional encounters could strengthen pharmacists’ contribution to chemsex prevention and harm reduction.”

Regarding the comment 5: “**The introduction would benefit from clarifying whether the KAP framework employed is based on a validated or previously used**

instrument, particularly given the risk of social desirability bias when assessing attitudes and practices related to stigmatized behaviors."

Author's response: We thank the reviewer for this comment. We have revised the Introduction to clarify that no validated chemsex-specific KAP framework currently exists in the pharmacy field, and that the KAP approach used in this study was newly developed for exploratory purposes (page 7, lines 151-158):

"A Knowledge–Attitude–Practice (KAP) study is particularly relevant in this context, as its exploratory approach allows to reveal what a target population knows, believes, and how they behave related to a health issue, thereby identifying gaps, misconceptions, and informational needs [23]. With KAP baseline data, prevention tools and policies can be tailored to actual deficits and attitudes with their determinants rather than assumed needs. To date, no chemsex-specific KAP framework has been applied in the pharmacy field, making this exploratory work original."

We also substantially revised the Methods section ("Questionnaire design and validation") to describe the development and validation process in detail, following established WHO and methodological guidelines (pages 10-11, lines 212–239). In addition, we now explicitly acknowledge the potential for social desirability bias and describe the measures taken to mitigate it, including anonymous, self-administered online data collection (page 8, lines 181-182, page 38, lines 647-652):

"Participation was voluntary and anonymous to reduce social desirability bias, and the questionnaire employed neutral, non-judgmental wording."

"Information bias may have occurred due to self-reported data, including recall bias regarding prior chemsex-related requests and social desirability bias, particularly for attitudes and perceived practices related to stigmatized behaviors. Participants may have overreported favorable attitudes toward prevention or perceived preparedness. To mitigate this risk, the questionnaire was anonymous, self-administered, and completed online, with no identifying information collected."

Regarding the comment 6: "**While the introduction appropriately situates chemsex within MSM communities, it would be helpful to explicitly justify how this framing translates into the professional scope of community pharmacists, particularly given the potential for assumptions about sexual orientation in pharmacy encounters.**"

Author's response: We thank the reviewer for this important clarification. We have revised the Introduction to explicitly state that, while chemsex is epidemiologically documented primarily within MSM communities, this framing does not imply that pharmacists should make assumptions about patients' sexual orientation. We clarify that the relevance of chemsex to community pharmacy practice lies in a behavior- and needs-based approach, through substance-related, sexual health–related, or harm reduction encounters, rather than identity-based targeting. This revision strengthens the ethical and professional rationale for pharmacists' involvement (pages 6–7, lines 141–148):

"In routine pharmacy practice, pharmacists interact with patients primarily through medication dispensing, harm reduction tools, and requests for information, rather than through discussions of sexual identity. Their potential role in chemsex prevention therefore lies in responding to substance-related, sexual health–related, or medication-related cues

(e.g., requests for HIV PrEP, post-exposure prophylaxis, STI treatment, or harm reduction equipment), irrespective of patients' sexual orientation. This behavior- and needs-based approach aligns with pharmacists' professional scope and ethical obligations, while avoiding assumptions about identity."

Regarding the comment 7: "**The construction of composite Knowledge, Attitude, and Practice scores requires demonstration of internal consistency. Reporting Cronbach's alpha for each subscale would strengthen confidence that the items measure coherent constructs.**"

<u>Author's response</u>: We thank the reviewer for this important methodological suggestion. We have now assessed the internal consistency of the composite Knowledge, Attitude, and Practice (perceived resource adequacy/need) scores using Cronbach's alpha, calculated on the full study sample. The Knowledge score showed acceptable internal consistency (α = 0.77), the Practice/perceived resource adequacy/need score showed good internal consistency (α = 0.81), and the Attitude score showed moderate internal consistency (α = 0.61), which is considered acceptable for an exploratory KAP survey assessing heterogeneous attitudinal constructs. These results have been added to the Methods and Results sections (page 13, lines 286-288; page 16, lines 341-343; page 23, lines 383-385; page 27, lines 438-439):

"These composite scores were constructed for descriptive and exploratory purposes. Internal consistency was assessed using Cronbach's alpha; however, the questionnaire was not intended as a fully psychometrically validated scale."

"The overall knowledge score ranged from 3 to 27, with a mean of 13.8 (SD = 4.4). Cronbach's alpha indicated acceptable internal consistency for this knowledge score (α = 0.77)."

"Participants' attitudes varied widely (mean attitude score = 9.6, SD = 2.9, range: 2–16). Internal consistency of the attitude score was moderate (α = 0.61), which is considered acceptable in exploratory KAP research."

"The perceived resource adequacy/need score had a mean of 9.58 (SD = 2.86, range: 5–16). Cronbach's alpha indicated good internal consistency for this score (α = 0.81)."

Regarding the comment 8: "**Recruitment via academic training supervisors may preferentially reach pharmacists who are more engaged with continuing education and university networks. As such, the sample may underrepresent more isolated practitioners, potentially limiting generalizability. Additionally, although the study is framed at the regional level, recruitment pathways appear primarily linked to Grenoble-based academic networks, which should be explicitly acknowledged as a limitation.**"

<u>Author's response</u>: We thank the reviewer for highlighting this important source of potential selection bias. We have revised the Limitations section to explicitly acknowledge that recruitment relied in part on pharmacy training supervisors affiliated with the Grenoble and Lyon faculties, both of which are now explicitly mentioned, which may have preferentially reached pharmacists more engaged in continuing education or university networks. We

now clearly state that this may limit the generalizability of the findings (pages 37–38, lines 629–638):

"Potential selection bias exists with the use of convenience and snowball sampling. Participants with a particular interest in sexual health or harm reduction may have been more likely to respond, as well as those more engaged with continuing education or university networks, since the initial sampling frame was derived from pharmacy training supervisors affiliated with the faculties of Grenoble and Lyon, which may have led to an overrepresentation of professionals connected to this academic network. Consequently, observed knowledge gaps may represent a conservative estimate relative to the broader community pharmacy workforce with findings that may not be fully generalizable to all community pharmacy professionals in the Auvergne-Rhône-Alpes region or elsewhere in France."

Regarding the comment 9: "**The use of forward stepwise regression, while common, has known limitations including coefficient instability and model overfitting. A theory-driven modeling strategy (e.g., hierarchical or full-model regression) may be more appropriate given the study's applied public health aims. If stepwise selection is retained, stronger justification and sensitivity analyses would be helpful.**»

Author's response: We thank the reviewer for this important methodological comment. We acknowledge the limitations of forward stepwise regression, including potential coefficient instability and overfitting. In our study, covariates were selected a priori based on theoretical relevance and prior literature, and stepwise selection was applied as an exploratory variable reduction strategy within this predefined set rather than as a purely data-driven approach. Given the descriptive and exploratory aims of this public health study, the regression analyses were intended to identify potential associations rather than to build predictive or causal models. We have clarified this rationale in the Methods section and explicitly acknowledged the limitations of stepwise modeling in the Discussion, emphasizing cautious interpretation of the findings (pages 13–14, lines 301–316; page 39, lines 663-666):

"To identify determinants associated with knowledge, attitude, and practice/need scores, separate multivariable linear regression models were fitted for each outcome. Covariates were selected a priori based on theoretical relevance and previous literature and included sociodemographic characteristics (items 1, 2, 4, 5: profession, pharmacy typology, gender, and age), source of awareness about chemsex (item 8), history of chemsex-related advice requests (item 20), and KAP scores. Years of practice (item 3) were excluded to avoid collinearity with age.
Covariates were selected a priori based on theoretical relevance and previous literature. Forward stepwise selection based on the Akaike Information Criterion (AIC) was then applied as an exploratory variable reduction strategy within this predefined set of covariates. Assumptions of linear regression (linearity, normality of residuals, homoscedasticity, and absence of multicollinearity) were assessed using residual plots. Regression coefficients (β), 95% confidence intervals (CI), and p-values were reported for all models. A two-sided significance threshold of $\alpha = 0.05$ was used, consistent with conventional practice in exploratory public health research. Given the exploratory nature of the study, the regression analyses were intended to identify potential associations rather than establish causal relationships."

"Moreover, the use of forward stepwise regression may have introduced model instability or overfitting, and the identified associations should therefore be interpreted as exploratory and hypothesis-generating rather than confirmatory."

Regarding the comment 10: "**The power calculation is briefly mentioned, but key parameters (assumed effect size, alpha level, and primary outcome) are not specified. Providing these details would improve transparency and reproducibility**.»

Author's response: We thank the reviewer for this comment. We agree that the term "power calculation" was imprecise for the objectives of this descriptive study. We have therefore revised the Methods section to clarify that the sample size was determined using a precision-based approach rather than hypothesis-driven power analysis. Specifically, the targeted sample size was calculated to estimate proportions with maximum variance (p = 0.50) using a two-sided 95% confidence interval and a predefined margin of error of ±7%, chosen as a pragmatic compromise between statistical precision and feasibility. As the study was exploratory and did not aim to test a specific hypothesis, no effect size or primary outcome was assumed. These details are now explicitly reported to improve transparency and reproducibility (page 9, lines 204–208):

"A minimum sample size of at least 196 participants was targeted to allow estimation of proportions with maximum variance (p = 0.50) with a two-sided 95% confidence interval and a margin of error of ±7% (chosen as a pragmatic compromise between acceptable precision and feasibility). The calculation was performed using the pwr package in R (version 4.2.1)."

Regarding the comment 11: "**The description of qualitative content analysis is brief. Additional detail regarding the analytic approach (e.g., inductive vs deductive coding, use of a coding framework, and strategies to enhance credibility) would strengthen methodological rigor.**"

Author's response: We thank the reviewer for this comment. We have expanded the Methods section to provide a detailed description of the qualitative content analysis, specifying the use of an inductive (conventional) content analysis approach, the absence of a predefined coding framework, the analytic steps followed, and strategies used to enhance credibility and transparency, including independent double coding and consensus procedures (page 13, lines 290–300):

"Qualitative free-text responses underwent conventional content analysis [27]. An inductive approach was applied, allowing categories to emerge directly from the data without a predefined coding framework. Coding was conducted iteratively through familiarization, open coding, and category development, and continued until category saturation was achieved. Meaning units were identified and grouped into categories; multiple categories could be assigned to a single response. Two researchers (M.L. and H.B.) independently coded all responses, with discrepancies resolved through discussion and consensus. Coding decisions were documented to ensure analytic transparency, and representative quotes were selected to illustrate categories. Descriptive frequencies of categories were calculated to identify prominent patterns in the data."

Regarding the comment 12: "**The term "practice score" may be misleading, as the items appear to assess perceived adequacy of resources and perceived needs rather than actual behaviors. Consider renaming to reflect the construct (e.g., 'perceived resource adequacy/need').** »

Author's response: We thank the reviewer for this important clarification. We agree that the original term "practice score" could be misleading, as the items primarily capture perceived adequacy of available resources and perceived ability to refer and support patients, rather than actual behaviors. To address this, we have renamed or added this outcome throughout the manuscript as "perceived resource adequacy/need score", and revised all relevant sections (Methods, Results, and Table 4) accordingly.

We retained the broader term "practices" when referring to the overall KAP framework of the study, but the specific score is now consistently labeled to reflect the construct it measures:

"It included 31 questions (25 closed, 6 open-ended), divided into four sections:
(i) Sociodemographic data (5 questions),
(ii) Chemsex knowledge (14),
(iii) Attitudes toward prevention (6)
(iv) Practices and perceived resource adequacy/ needs (6)."

"Perceived resource adequacy/need score (capturing the perceived adequacy of available resources and perceived practical ability to refer and support patients): Sum of items 24, 25, 29, and 30 (Likert items scored 0–4; specific scoring for item 24 based on perceived adequacy of current available resources)."

Regarding the comment 13: "**A substantial proportion incorrectly endorsed cannabis/hallucinogens as chemsex-related substances, suggesting a broad "drug use during sex" interpretation rather than the more specific set of substances commonly emphasized in chemsex harm profiles. This reinforces the need for clear definitional training.** »

Author's response: We thank the reviewer for this comment and have addressed it in the Discussion section as follows (pages 32-33, lines 510-515):

"Furthermore, the substantial proportion of participants who incorrectly endorsed cannabis or hallucinogens as chemsex-related substances suggests a broader interpretation of chemsex as "drug use during sex", rather than the more specific set of substances commonly emphasized in chemsex-related harm profiles. This misclassification underscores the need for clearer definitional and educational training."

Regarding the comment 14: "**Male gender was associated with higher knowledge scores. The paper should avoid essentialist explanations and consider alternative mechanisms (differential exposure to information, confidence in self-assessment, workplace role differences).** »

Author's response: We thank the reviewer for this important comment. We agree that essentialist interpretations should be avoided. We have revised the Discussion section to clarify that the observed association between male gender and higher knowledge scores

does not reflect inherent gender differences. We now explicitly discuss alternative explanatory mechanisms, including differential exposure to chemsex-related information, professional encounters, self-assessment confidence, and potential workplace role differences, and emphasize the need for cautious interpretation given the cross-sectional design (page 333, lines 525–533):

“The association between male gender and higher knowledge scores should be interpreted cautiously and does not imply inherent gender-based differences. Several alternative mechanisms may account for this finding, including differential exposure to information on chemsex through professional encounters, continuing education, or informal networks; greater likelihood of prior contact with chemsex-related situations; or differences in self-perceived confidence when responding to knowledge items. Workplace role distribution and task allocation within pharmacies may also influence exposure to sexual health or harm reduction topics. Given the cross-sectional and self-reported nature of the data, residual confounding and reporting bias cannot be excluded.”

Regarding the comment 15: “**Given the stated inclusion of open-ended items and content analysis, the Results should report qualitative themes and provide representative quotes (with anonymized identifiers). If qualitative findings are minimal, the manuscript should clarify the role of open-ended data and avoid overstating mixed-methods contributions.** »

Author’s response: We thank the reviewer for this comment. In response, we have revised the Results section to explicitly report findings from the qualitative content analysis of open-ended items. We now present the inductively derived categories that emerged from the data and include representative verbatims with anonymized identifiers to illustrate each category (page 13, lines 290-300, lines 359-365; page 17, lines 355-357; page 23, lines 386-400; page 24, lines 405-420; pages 28-29, lines 447-487; Tables 2–4).

In addition, we clarified the role of the qualitative component as complementary to the quantitative findings, aiming to contextualize and deepen the interpretation of pharmacists’ knowledge gaps, attitudes, and perceived needs rather than to generate standalone qualitative theory. These revisions ensure that the qualitative data are transparently presented and substantiate the mixed-methods nature of the study (page 37, lines 621-624):

“The use of a structured KAP framework, combined with both quantitative analyses and qualitative content analysis of open-ended responses, offers a comprehensive and nuanced understanding of current gaps, perceptions, and needs relevant to chemsex prevention in community pharmacy settings.”

Regarding the comment 16: “**The Discussion should explicitly highlight the low recognition of injection (“slam”) as part of chemsex (18.1%). Given the elevated acute harms and blood-borne infection risks associated with injection, this knowledge gap may limit pharmacists’ ability to provide harm-reduction counseling when dispensing sterile equipment.** »

Author’s response: We thank the reviewer for this important comment. We have revised the Discussion to explicitly highlight the low recognition of injection (“slam”) as part of chemsex and to emphasize its implications for pharmacy-based harm reduction. The

revised text now discusses how this knowledge gap may limit pharmacists' ability to provide appropriate counseling when dispensing sterile injection equipment, despite the elevated risks of acute harms and blood-borne infections associated with injecting practices. This addition strengthens the link between pharmacists' knowledge gaps and their potential role in preventing chemsex-related harms (page 34, lines 541–548):

"Regarding knowledge gaps with direct implications for harm-reduction counseling, the low recognition of injection ("slam") as part of chemsex (18.1%) is particularly concerning. This limited awareness may restrict pharmacists' ability to deliver appropriate harm-reduction messages when dispensing sterile injection equipment, despite the elevated risks of acute toxicity and blood-borne infections associated with injecting practices. Given that community pharmacies already serve as a key access point for sterile equipment, insufficient recognition of slam may result in missed opportunities for targeted counseling and risk reduction."

Regarding the comment 17: "**The Discussion should address the apparent discrepancy between low demonstrated knowledge of drug–drug interactions/reference resources and relatively high self-reported confidence in referral capacity. This may indicate that professionals conceptualize chemsex primarily as a psychosocial issue requiring referral, potentially overlooking medication-safety responsibilities at the point of dispensing. Training could therefore integrate both referral pathways and practical medication-safety decision support.** »

Author's response: We thank the reviewer and have added a dedicated discussion paragraph to explicitly address the discrepancy between low demonstrated knowledge of drug–drug interactions/reference resources and high self-reported referral capacity (pages 35–36, lines 582–589):

"The discrepancy between low demonstrated knowledge of drug–drug interactions or reference resources and high self-reported referral capacity further suggests that professionals may conceptualize chemsex primarily as a psychosocial issue requiring referral, potentially overlooking medication-safety responsibilities at the point of dispensing. Training initiatives could therefore benefit from integrating both referral pathways and practical medication-safety decision support. In addition, social desirability bias may have contributed to overreporting perceived referral capacity."

Regarding the comment 18: "**Recruitment through academic training-supervisor networks and snowballing may have overrepresented pharmacists connected to university settings and continuing education. This may limit generalizability and could mean observed knowledge gaps represent a conservative estimate relative to the broader community pharmacy workforce.** »

Author's response: We thank the reviewer for this comment, which is also related to comment n°8. As noted in our response to comment n°8, we have revised the Limitations section. We now explicitly acknowledge that recruitment partially relied on pharmacy training supervisors affiliated with the Grenoble and Lyon faculties, which may have preferentially reached pharmacists more engaged in continuing education or university networks (pages 37–38, lines 629–638):

“Potential selection bias exists with the use of convenience and snowball sampling. Participants with a particular interest in sexual health or harm reduction may have been more likely to respond, as well as those more engaged with continuing education or university networks, since the initial sampling frame was derived from pharmacy training supervisors affiliated with the faculties of Grenoble and Lyon, which may have led to an overrepresentation of professionals connected to this academic network. Consequently, observed knowledge gaps may represent a conservative estimate relative to the broader community pharmacy workforce with findings that may not be fully generalizable to all community pharmacy professionals in the Auvergne-Rhône-Alpes region or elsewhere in France.”

Regarding the comment 19: “**The models explain a modest proportion of variance for attitude and perceived needs, suggesting that unmeasured factors (e.g., stigma-related beliefs, professional norms, prior training experiences) may be influential. This should be acknowledged as a limitation.**”

Author’s response: We thank the reviewer for this comment. We have expanded the limitations section to interpret the implications of the moderate explained variance observed in the regression models. We now clarify that unmeasured factors may be influential, and we discuss how this limits causal interpretation (page 39, lines 656–666):

“The multivariable regression models explained a moderate variance for knowledge (35.8%) but less for attitudes (11%) and perceived resource adequacy/need (8%). This suggests that while individual characteristics and prior exposure to chemsex-related situations partly explain knowledge levels, attitudes and perceived practices are likely shaped by additional unmeasured factors. These may include personal beliefs, perceived professional role boundaries, prior trainings, moral or cultural representations of sexuality and substance use, prior informal experiences with chemsex-related situations, organizational context, and perceived institutional support. Moreover, the use of forward stepwise regression may have introduced model instability or overfitting, and the identified associations should therefore be interpreted as exploratory and hypothesis-generating rather than confirmatory.”

General comment:

During careful proofreading of the submitted manuscript, we identified an omission in Table 2, where one line was inadvertently missing in the originally submitted file. This has now been corrected in the revised version (line: “Self-declared knowledge of a database for drug interactions in chemsex”). We apologize for this oversight.

VERSION 2 - REVIEW

**Reviewer**	**3**
**Name**	**Khan, Mohammad Niaz Morshed**
**Affiliation**	**International Centre for Diarrhoeal Disease Research Bangladesh, Infectious Disease Division**

Date **14-Feb-2026**

COI

General comments

I thank the authors for their careful revision of the manuscript. The authors have addressed the main concerns raised in the initial review and have improved the clarity, transparency, and overall quality of the study.

The inclusion of qualitative findings from open-ended responses resolves the previous inconsistency between the stated mixed-methods design and the reported results. The qualitative analysis, including themes and example quotations, adds useful context and helps explain pharmacists' perceptions, barriers, and training needs.

The authors have also improved the statistical reporting by providing Cronbach's alpha values for the Knowledge, Attitude, and Practice scores. This addition strengthens confidence that these scores measure meaningful and internally consistent constructs.

Overall, the manuscript is now clearer, more coherent, and methodologically stronger.

Specific comments

1. The authors have explained their use of forward stepwise regression as an exploratory approach. While theory-based modelling is generally preferred, the use of stepwise regression is acceptable in this exploratory study, especially since the authors have acknowledged its limitations.

2. The Cronbach's alpha for the attitude score is moderate (0.61). This is acceptable for exploratory research. However, the authors may briefly acknowledge this moderate reliability in the Discussion section and note that future studies should further validate this scale.

3. The authors have clarified the sampling strategy and its limitations, including recruitment through academic and professional networks. This improves the interpretation of the findings and appropriately limits generalization.

Recommendation: The authors have adequately addressed the major concerns raised in the initial review. The manuscript now presents a clear and methodologically sound exploratory study with important implications for public health practice.

VERSION 2 - AUTHOR RESPONSE

Point by point response to Reviewer 3:

Regarding the general comments: "I thank the authors for their careful revision of the

manuscript. The authors have addressed the main concerns raised in the initial review and have improved the clarity, transparency, and overall quality of the study.
The inclusion of qualitative findings from open-ended responses resolves the previous inconsistency between the stated mixed-methods design and the reported results. The qualitative analysis, including themes and example quotations, adds useful context and helps explain pharmacists' perceptions, barriers, and training needs.
The authors have also improved the statistical reporting by providing Cronbach's alpha values for the Knowledge, Attitude, and Practice scores. This addition strengthens confidence that these scores measure meaningful and internally consistent constructs.
Overall, the manuscript is now clearer, more coherent, and methodologically stronger."

Author's response: We thank Reviewer 3 for this positive, constructive, and detailed feedback. We are pleased that the revisions have improved the clarity, coherence, and methodological rigor of the manuscript, and that the inclusion of qualitative findings and additional statistical analyses has addressed the initial concerns.

Regarding the specific comment 1: "The authors have explained their use of forward stepwise regression as an exploratory approach. While theory-based modelling is generally preferred, the use of stepwise regression is acceptable in this exploratory study, especially since the authors have acknowledged its limitations."

Author's response: We thank the reviewer for this comment. We are pleased that the justification for the use of forward stepwise regression as an exploratory approach is considered acceptable. As suggested, we have ensured that its limitations are clearly acknowledged in the manuscript and that the results are interpreted cautiously as exploratory and hypothesis-generating.

Regarding the specific comment 2: "The Cronbach's alpha for the attitude score is moderate (0.61). This is acceptable for exploratory research. However, the authors may briefly acknowledge this moderate reliability in the Discussion section and note that future studies should further validate this scale."

Author's response: We thank the reviewer for this relevant suggestion. We have now explicitly acknowledged in the Discussion section that the internal consistency of the Attitude score was moderate (Cronbach's $\alpha = 0.61$), which is acceptable in an exploratory context but indicates the need for further refinement and psychometric validation in future studies (page 32, lines 667–673):

"Finally, although internal consistency of the composite scores was acceptable, the questionnaire was newly developed and not intended as a fully psychometrically validated instrument. The Attitude score showed moderate reliability (Cronbach's $\alpha = 0.61$), which is acceptable for exploratory research but suggests that further refinement and validation are warranted. Further studies should aim to confirm these findings and to conduct

comprehensive psychometric validation of chemsex-specific KAP tools for pharmacy professionals."

Regarding the specific comment 3: "The authors have clarified the sampling strategy and its limitations, including recruitment through academic and professional networks. This improves the interpretation of the findings and appropriately limits generalization."

Author's response: We appreciate the reviewer's recognition that the revised manuscript provides a clearer description of the sampling strategy and its limitations. We are glad that these additions have strengthened the interpretation of the findings and appropriately framed the limits of generalizability.

Regarding the recommendation: "The authors have adequately addressed the major concerns raised in the initial review. The manuscript now presents a clear and methodologically sound exploratory study with important implications for public health practice."

Author's response: We thank the reviewer for this positive and encouraging assessment. We are pleased that the revisions have adequately addressed the major concerns and that the manuscript is now considered clear and methodologically sound, with relevant implications for public health practice.
